# Antibiotic-Induced Shifts in Fecal Microbiota Density and Composition during Hematopoietic Stem Cell Transplantation

**DOI:** 10.1128/IAI.00206-19

**Published:** 2019-08-21

**Authors:** Sejal Morjaria, Jonas Schluter, Bradford P. Taylor, Eric R. Littmann, Rebecca A. Carter, Emily Fontana, Jonathan U. Peled, Marcel R. M. van den Brink, Joao B. Xavier, Ying Taur

**Affiliations:** a Infectious Disease Service, Department of Medicine, Memorial Sloan Kettering, New York, New York, USA; b Immunology Program, Sloan Kettering Institute, Memorial Sloan Kettering, New York, New York, USA; c Center for Microbes, Inflammation and Cancer, Memorial Sloan Kettering, New York, New York, USA; d Computational Biology Program, Sloan Kettering Institute, Memorial Sloan Kettering, New York, New York, USA; e Adult Bone Marrow Transplant Service, Department of Medicine, Memorial Sloan Kettering, New York, New York, USA; f Weill Cornell Medical College, New York, New York, USA; University of Michigan—Ann Arbor

**Keywords:** antibiotics, commensal anaerobes, hematopoietic cell transplantation, microbiome, systems biology

## Abstract

Dramatic microbiota changes and loss of commensal anaerobic bacteria are associated with adverse outcomes in hematopoietic cell transplantation (HCT) recipients. In this study, we demonstrate these dynamic changes at high resolution through daily stool sampling and assess the impact of individual antibiotics on those changes. We collected 272 longitudinal stool samples (with mostly daily frequency) from 18 patients undergoing HCT and determined their composition by multiparallel 16S rRNA gene sequencing as well as the density of bacteria in stool by quantitative PCR (qPCR). We calculated microbiota volatility to quantify rapid shifts and developed a new dynamic systems inference method to assess the specific impact of antibiotics. The greatest shifts in microbiota composition occurred between stem cell infusion and reconstitution of healthy immune cells. Piperacillin-tazobactam caused the most severe declines among obligate anaerobes. Our approach of daily sampling, bacterial density determination, and dynamic systems modeling allowed us to infer the independent effects of specific antibiotics on the microbiota of HCT patients.

## Abstract (precis)

Dramatic microbiota changes and loss of commensal anaerobic bacteria are associated with adverse outcomes in hematopoietic cell transplantation (HCT) recipients. In this study, we demonstrate these dynamic changes at high resolution through daily stool sampling and assess the impact of individual antibiotics on those changes.

## INTRODUCTION

Patients with a range of hematologic malignancies can be treated and potentially cured by hematopoietic cell transplantation (HCT). Prior to HCT, chemotherapy and/or total body irradiation is performed to deplete the cancerous cells. These treatments, combined with simultaneous antibiotic administration, compromise immune defenses, damage the mucosal epithelium, and deplete the native intestinal microbiota, facilitating the emergence of antibiotic-resistant organisms and increasing the risk of infections (1, 2). Microbiome studies using fecal samples collected from allogeneic HCT (allo-HCT) patients have previously revealed that patients experience a severe reduction in the relative abundance of commensal bacteria over the course of treatment. This loss can result in blooms of potentially pathogenic microbial species (3), leading to downstream complications such as infections and graft-versus-host disease (GVHD) (4–6). In particular, the loss of obligate anaerobic commensal bacteria such as *Clostridia* and *Bacteroidetes* negatively influences HCT outcomes, as shown in both animal models and humans (4, 7, 8).

Yet the relative degree and manner in which various antibiotics and conditioning regimens contribute to microbiota disruption is still not well described. In previous studies examining the changes in the microbiota of HCT patients, stool samples were collected either approximately once per week or at a limited number of time points (3–5, 9, 10). Though these studies helped to form the foundation of our current understanding of microbiota disruption during allo-HCT, we posit that a more frequent stool sample collection scheme, combined with dynamic modeling, would be beneficial for providing a higher-resolution view of changes in microbiota composition over time and discerning individual antibiotic effects. Additionally, stool samples from many previous studies were characterized only in terms of relative abundance using 16S sequencing, which does not allow quantitative calculations of species loss (11) and, therefore, could potentially hamper attempts to quantitatively assess the effects of antibiotics on the microbiota.

In this study, we collected near-daily stool samples from 18 HCT recipients, for which we analyzed total species abundance by combining 16S sequencing in conjunction with quantitative PCR (qPCR) of the 16S gene. We leveraged classical models of microbial growth with Bayesian regression techniques to quantify the impact of specific classes of antibiotics on anaerobic microbes representative of a healthy gut. Importantly, our model can be extrapolated to clinically guide sequential drug treatments that minimize detrimental effects on commensal bacteria. Our results reveal how important commensal anaerobic microbial species are lost during HCT.

## RESULTS

### Description of study population and biospecimens.

**FIG 1:**
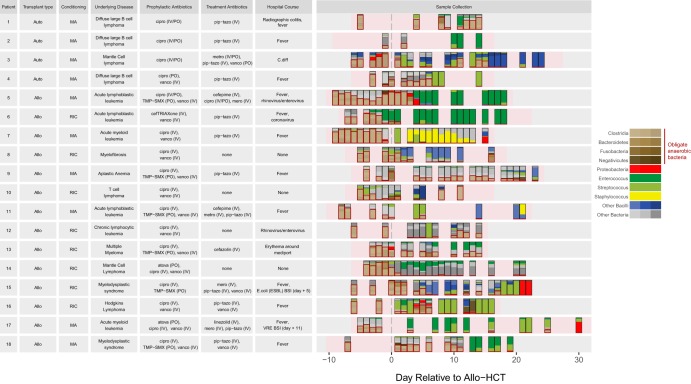
Clinical characteristics of all 18 HCT patients. The right panel depicts the timing of stool collection during the course of transplantation (rectangles), relative to hematopoietic cell infusion (day 0), for each patient. Stacked colors represent each sample’s microbiota composition (based on 16S sequencing). Boxes are drawn around the anaerobes. Pink shading represents times of inpatient hospitalization. Abbreviations: Auto, autologous; Allo, allogeneic; RIC, reduced intensity conditioning; MA, myeloablative; IV, intravenous; PO, oral; cipro, ciprofloxacin; metro, metronidazole; pip-tazo, piperacillin-tazobactam; mero, meropenem; vanco, vancomycin; TMP-SMX, trimethoprim-sulfamethoxazole; C. diff, Clostridium difficile; BSI; bloodstream infection; VRE, vancomycin-resistant enterococci; atova, atovaquone.

Our cohort consisted of 18 patients who underwent auto- or allo-HCT at Memorial Sloan Kettering Cancer Center (MSKCC) between July 2015 and January 2016. Clinical characteristics and stool microbiome data for each patient are shown in Fig. 1. Patients underwent different types of HCT for a variety of conditions; 14 patients underwent allo-HCT and 4 underwent auto-HCT. The duration of transplant hospitalization ranged from 20 to 38 days, during which antibiotics were given for both prophylactic and treatment purposes. Throughout this period, we sought to collect fecal samples on a daily basis. A total of 272 samples were collected from the 18 patients (77% of a total of 352 inpatient hospital days). Of those samples, 236 (87%) yielded 16S amplicons that could be sequenced.

**FIG 2:**
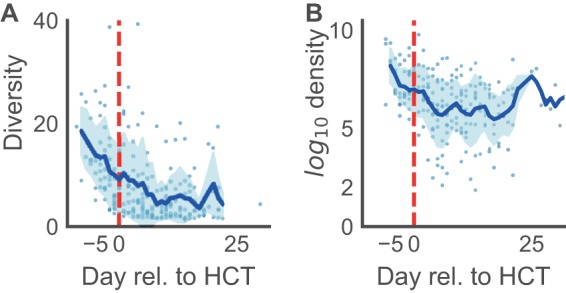
Microbiota changes in diversity and density during HCT. (A) During conditioning, before hematopoietic cell transfusion (day 0, red dashed line), the community diversity of the microbiota in both allo- and auto-HCT patients declined rapidly. (B) Similarly, the bacterial density declined, plotted as the total number of bacterial cells per gram of stool, and only mild recovery of cell counts was observed toward the latest days of hospitalization (and there, mostly in allo-HCT patients) (see Fig. S1 in the supplemental material).

**FIG 3:**
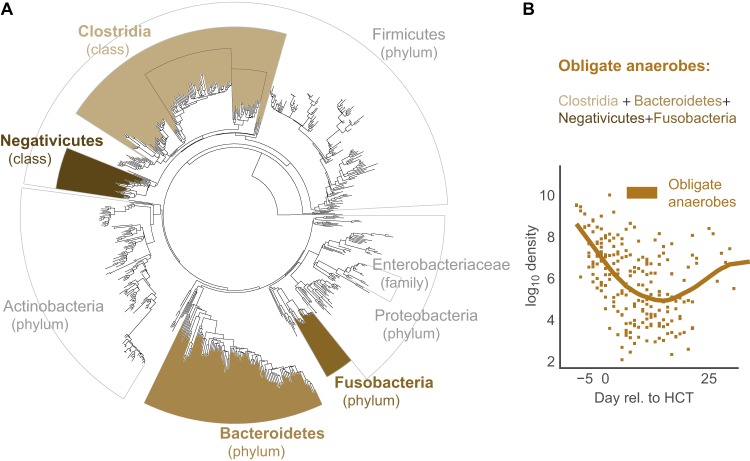
Obligate anaerobe grouping. (A) Bacterial phylogenetic tree indicating in brown the groups we classified as commensal anaerobes. (B) Average commensal anaerobe density across all patients. The line indicates mean values per day using locally weighted scatterplot smoothing (22).

Consistent with previous findings (3, 5), all patients presented with highly diverse microbial populations prior to HCT with species compositions that included diverse healthy anaerobic microbes; subsequent antibiotic administration caused large-scale changes to the intestinal microbiota with decreases specifically noted in the diversity of the intestinal population and of the bacterial population density as a whole (Fig. 1 and 2). This observation was noted in both allo-HCT and auto-HCT patients (see Fig. S1 in the supplemental material), coinciding with completion of pretransplant conditioning and administration of broad-spectrum antibiotics around day 0 (Fig. S2). We also characterized stool consistency as either formed, semiformed, or liquid and found that patients with diarrhea (liquid stool) had lower bacterial counts (by 16S qPCR) in their respective stool samples (Fig. S3). We focused on the obligate anaerobic bacterial species that fall in the classes *Clostridia*/*Negativicutes* and the phyla *Fusobacteria*/*Bacteroidetes* (Fig. 3A) and noted a sharp decline in these bacterial populations post-HCT (Fig. 3B).

### Subject-level microbiome changes.

**FIG 4:**
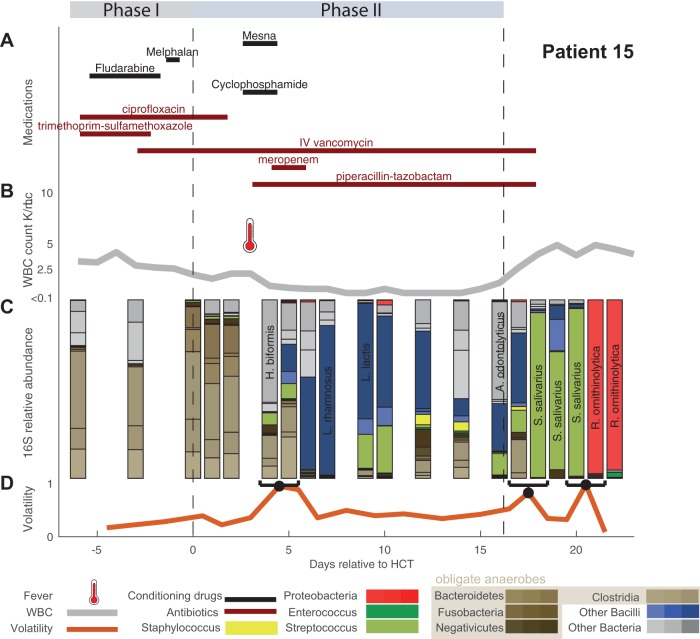
Timeline over the course of transplantation for a single HCT patient (patient 15). (A) Antibiotic administration and chemotherapy regimen during conditioning (phase I), post-HCT neutropenia before engraftment (phase II), and postengraftment. (B) White blood cell (WBC) counts across treatment with fever (thermometer) are indicated. (C) Relative abundances by 16S sequencing grouped at the indicated taxon level during this patient’s HCT admission were collected almost daily. On day +3 the patient had a fever and received broad-spectrum antibiotics as a result. (D) Volatility quantifies the rate of change in microbiota composition across adjacent time points. A. odontolyticus, Actinomyces odontolyticus; H. biformis, Holdemanella biformis; L. lactis, Lactococcus lactis; L. rhamnosus, Lactobacillus rhamnosus; R. ornithinolytica, Raoultella ornithinolytica; S. salivarius, Streptococcus salivarius.

A single subject’s timeline (patient 15) is presented in Fig. 4, showing medications and treatments, clinical data, and intestinal microbiome composition (Fig. 4A to C). Data for all other patients are shown in Fig. S4. We observed successive alterations of the intestinal microbiota population in each patient over the course of transplantation. These dynamic changes seemed to correspond with specific changes in antibiotic administration. The loss of obligate anaerobic bacteria appeared to coincide more with the administration of certain types of antibiotics. Anaerobic microbes seemed relatively spared in some patients during periods when they remained only on prophylactic antibiotics (i.e., intravenous [i.v.] vancomycin or ciprofloxacin). In these 18 patients, we observed two patients with bloodstream infections, which were preceded by intestinal expansion of the corresponding pathogen (Escherichia coli in patient 5 and vancomycin-resistant Enterococcus faecium [VRE] in patient 17) (Fig. S4).

To quantify day-to-day community shifts, we assessed the compositional volatility of the microbiota between daily intervals, reflecting the overall degree of compositional change over time (Fig. 4D and Fig. S4). In our patients, microbiota volatility was, on average, highest immediately following transplant (Fig. S5).

### Antibiotic-induced loss of obligate anaerobic bacteria.

**FIG 5:**
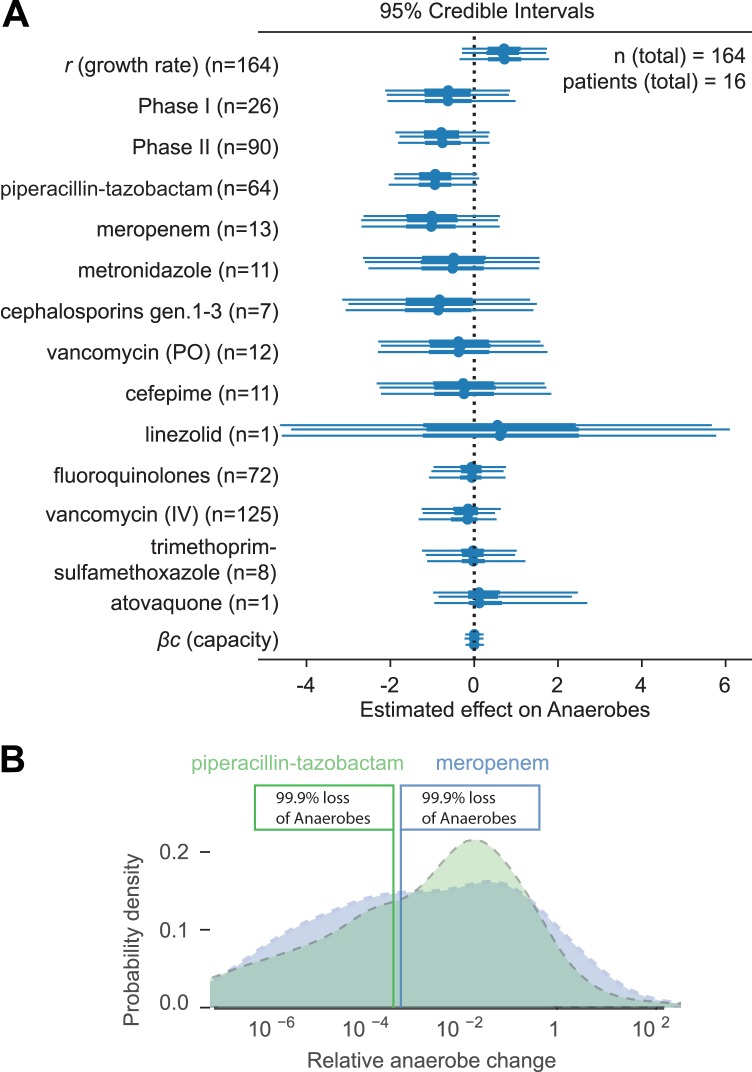
Specific antibiotic effects in HCT patients. (A) Posterior parameter estimates from Bayesian linear regression of our model of antibiotic effects on obligate anaerobes. The 95% credibility intervals from three independent Markov chain Monte Carlo traces with no-U-turn sampling are shown. (B) Distributions of predicted loss of anaerobes due to piperacillin-tazobactam and meropenem courses typical for our patient cohort. See Materials and Methods for details.

Our model of microbial growth of obligate anaerobic bacteria identified piperacillin-tazobactam and meropenem as independently having the most detrimental impact on obligate anaerobes. Additionally, our model indicated some negative effect of metronidazole, oral vancomycin, and cephalosporins (generations 1 to 3), albeit with limited credibility, whereas fluoroquinolones, vancomycin (i.v.), and trimethoprim-sulfamethoxazole had very little impact (Fig. 5A and Table S1). Furthermore, our model identified loss of obligate anaerobic bacteria when a patient experienced post-HCT neutropenia before neutrophil engraftment (phase II), in addition to the effects of antibiotics. The model did not infer an exponential-growth-limiting effect of the obligate anaerobe community on itself (capacity). We were able to qualitatively capture the anaerobe dynamics of each patient’s time course, including major inflection points, by simulating data for each patient forward in time, starting with the first observed density of commensal anaerobes (Fig. S6).

We then predicted the effect of entire courses of piperacillin-tazobactam and meropenem as they occurred among our patients, i.e., including the effects of the period when they were administered (phase I, phase II, or thereafter) as well as of coadministered antibiotics (Fig. 5B; see also Materials and Methods). Due to differences in the duration of administrations and variation in coadministered antibiotics during these courses, the predicted loss of anaerobes was also variable (e.g., a longer course would yield a larger total loss of anaerobes). Yet, importantly, our model estimated that among our patients, courses with meropenem and with piperacillin-tazobactam each lead to >99% loss of obligate anaerobic bacteria (Fig. 5B).

## DISCUSSION

Under normal circumstances, the human intestinal microbiota is relatively stable over time and largely consists of obligate anaerobic bacteria (12). In contrast to this norm, our study shows dramatic day-to-day shifts in bacterial density and microbiota composition for HCT patients undergoing antibiotic treatment. Given the frequent administration of broad-spectrum antibiotics and the impact of HCT on immune defenses, mucosal epithelial integrity, and dietary intake, these extreme shifts in microbiota composition are perhaps unsurprising. In this study, our goal was to contextualize these compositional changes in the microbiota in terms of response to specific antibiotics (13). We believe that a precise understanding and appreciation of antimicrobial influences on the microbiota can help inform antibiotic decision making within the clinical setting and protect against microbiota disruption and pathogen invasion. Maintaining intestinal diversity in HCT patients has major clinical implications, including offering protection from developing GVHD and bloodstream infections and improving overall survival and transplant-related mortality (3–5). Our study is a first step toward the goal of minimizing unintended collateral damage to the commensal microbiota through the improved use of antibiotics.

Here, we show that patients can differ in terms of the rapidity, magnitude, and quality of microbiota alterations, which likely reflect differences in baseline microbiota composition, exposure to antibiotics, and degrees of immune compromise and epithelial damage. The frequent stool sampling coupled with 16S qPCR and detailed clinical data allowed us to now quantify the impact of specific antibiotics on microbiota composition. In our modeling approach, we incorporated absolute measures of 16S abundance and therefore were able to study microbiome dynamics as rate changes. This would be impossible with 16S relative abundance, and the resulting covariance bias could make it impossible to disentangle loss of anaerobes from increases in other taxa (14). In some instances, our goal of daily stool collection was not well met, leading to interval censoring, a period of time where the microbiota composition is unknown.

We decided to analyze obligate anaerobic bacteria together in a single group (*Clostridia*, *Bacteroidetes*, *Negativicutes*, and *Fusobacteria*), given their ties to healthy immunity and colonization resistance (4, 8, 10). The premise that obligate anaerobic bacteria represented the vast majority of the normal colonic flora and conferred colonization resistance (15, 16) is rooted in premicrobiome observations using culture-based methods, some as far back as 50 years ago (17). Several studies have showcased their critical role in maintaining intestinal immune homeostasis (16, 18, 19) and an association with good clinical outcomes (20, 21). Admittedly, it is not known to what degree being anaerobic approximates a truly beneficial microbiota. That said, by attempting to combine and study obligate anaerobic bacteria as we did, we were able to better align our results with existing clinical knowledge (22); our model identified piperacillin-tazobactam and meropenem as major causes of obligate anaerobe loss, while metronidazole, which was administered only rarely, did not have an equally strong effect.

Our approach also demonstrated a degree of anaerobic impact from cephalosporins (generations 1 to 3). Although not traditionally used to treat anaerobic infections, ceftriaxone and cefazolin have been shown to have some activity against *Clostridium* spp. (23). The killing potential of cephalosporins could also be explained by confounding factors such as the concurrent administration with specific conditioning treatments during phase I that were not included explicitly in our model (patients 6 and 13) (see Fig. S4 in the supplemental material).

Indeed, we observed significant anaerobic loss during the time window of phase II, i.e., postconditioning and HCT infusion but prior to neutrophil engraftment day. Independent of antibiotic effects, these results may encapsulate direct HCT treatment- and immune suppression-related effects, including the cytotoxic effects of chemotherapy and/or radiation. We hypothesize that these factors contribute to microbiota disruption, either directly by the killing of anaerobic bacteria or indirectly by impairing host mucosal mechanisms for intestinal homeostasis.

HCT exposes the intestinal microbiota to a wide variety of environmental changes and creates a complex ecosystem that is difficult to model. However, despite this being a pilot study of HCT patients, we feel that our model performed well and provided promising results largely consistent with our clinical impressions. Discerning the individual effects of different antibiotics and chemotherapy on the microbiota was a challenge as simultaneous drug administrations are common, but we are encouraged by our model estimations for individual antibiotic effects. Our complex patient population and small sample size meant that some of our results consisted of wide credibility intervals, which estimate population parameters with lower precision. Our model therefore predicted the time courses of patients qualitatively, capturing inflection points of major anaerobe loss rather than predicting time courses with high quantitative accuracy. To bypass these potential limitations, we will continue to accumulate high-frequency, quantitative microbiome data in conjunction with detailed clinical metadata to better predict the individual effects of each drug.

Understanding collateral damage to the microbiota is relevant to prevent not only microbiome dysbiosis-related disease but also the rise of antibiotic-resistant pathogens (13, 24). A better understanding of the dynamics that render a complex microbiota permissive to pathogen expansion has the potential to shape and improve basic principles of antibiotic stewardship. Clinicians should be aware of antibiotics that have more potential to disrupt the microbiota (i.e., piperacillin-tazobactam) when prescribing them, shorten the duration of these antibiotics when feasible, and consider microbiota-protective therapies such as a fecal microbiota transplantation in extreme cases to protect colonic microbiota integrity (25).

## MATERIALS AND METHODS

### Study patients and fecal sample collection.

We followed 18 adult patients undergoing auto-HCT or allo-HCT at Memorial Sloan Kettering Cancer Center (MSKCC) from July 2015 to January 2016. There were 7 female and 11 male patients; their ages ranged from 40 to 75 years. Fecal samples were collected longitudinally from each patient during their transplant hospitalization using a prospective institutional fecal biospecimen collection protocol (described previously [3]). For the majority of patients, daily collection began at the start of pretransplant conditioning (7 to 10 days before hematopoietic cell infusion) and continued until discharge, typically a month after HCT. The study protocol was approved by the MSKCC institutional review board; informed consent was obtained from all subjects prior to sample collection.

### Transplantation practices.

At MSKCC, antimicrobial prophylaxis is given routinely to patients undergoing HCT. Subjects undergoing either auto- or allo-HCT are given oral (*per os* [p.o.]) ciprofloxacin 2 days prior to hematopoietic cell infusion as prophylaxis against Gram-negative bacterial infections. Allo-HCT recipients are also given intravenous (i.v.) vancomycin as prophylaxis against viridans-group streptococci (26). Antibiotic prophylaxis against Pneumocystis jirovecii pneumonia was generally administered using either trimethoprim-sulfamethoxazole, aerosolized pentamidine, or atovaquone; the time at which prophylaxis was initiated (during conditioning or after engraftment, defined as an absolute neutrophil count of ≥500 neutrophils/mm^3^ for three consecutive days) varied. In the event of a new fever during times of neutropenia, patients were usually started on empirical antibiotics, such as piperacillin-tazobactam, cefepime, or meropenem.

### Sample analysis of microbial population composition.

Sample DNA was extracted and purified, and the V4-V5 region of the 16S rRNA gene was amplified with PCR using modified universal bacterial primers. Sequencing was performed using a Illumina MiSeq platform (27) yielding paired-end reads with lengths up to 250 bp. These reads were assembled, processed, filtered for quality, and grouped into operational taxonomic units of 97% similarity using the UPARSE pipeline (28). Taxonomic assignment to species level was performed using nucleotide BLAST (Basic Local Alignment Search Tool) (29), with the National Center for Biotechnology Information RefSeq (refseq_rna) as the reference database (30). We determined the copy number of 16S rRNA genes per gram of stool for each sample by quantitative PCR (qPCR) on total DNA extracted from fecal samples (31–33). We assessed microbially diverse populations using the inverse Simpson index (for additional experimental details and microbiome data availability, see Methods in the supplemental material; all data used in this study are available as an Excel file).

### Analytic approach.

We developed and employed a metric of compositional volatility to quantify the rate of overall change in microbiota composition across adjacent samples in time. The metric assesses overall community change by calculating the Manhattan distance between microbiota compositions and ranges between 0 and 1. It can be interpreted as the fraction of community turnover when pairs of consecutive samples within a single patient are compared. The volatility, *V*, for the community grouped at a taxonomic level, *I*, was determined by the following expression:

$$V\left(t+\Delta t/2\right)=\frac{1}{2\Delta t}\underset{i\in I}{\sum }\left|X_{i}\left(t+\Delta t\right)-X_{i}\left(t\right)\right|$$

where Δ*t* is the time in days between the consecutive samples and *X_i_*(*t*) is the relative abundance of taxon *i* at time *t*. The inverse time scaling allows volatility to be interpreted as average change in composition over time. We calculated volatility using relative abundances of microbes taxonomically grouped at the genus level. Theoretically, the most volatile points (*V* ≈ 1) would correspond to complete microbiota replacements between two time-adjacent samples, whereby previously abundant genera would be completely replaced by different genera.

The total abundances of anaerobes were calculated by multiplying the summed relative abundances of obligate anaerobic taxa obtained by 16S sequencing (see Methods in the supplemental material for protocols and details) with the total copy numbers of 16S genes obtained via qPCR. To estimate the effects of different antibiotics on specific microbial groups, we calculated the log-difference of absolute anaerobe cell counts per gram of stool (wet weight) between two samples (deltas) which were at most 2 days apart and within the first hospitalization. We used Bayesian regression techniques to parameterize a model of the logistic growth of the obligate anaerobe community, used similarly by Stein et al. (32). Antibiotic effects on bacterial reproduction or death were modeled as independently modifying the anaerobe population growth rate. Part of the model design included choices on how to aggregate clinical interventions, and we chose a hierarchical approach. To account for potential HCT-related microbiota damage, we aggregated pre-HCT cytotoxic conditioning treatments as a single indicator variable, named phase I, and the period of post-HCT neutropenia preengraftment with another indicator variable, phase II. While these indicator variables are intended to capture additional effects on the microbiota independent of the administration of antibiotics, often they coincided with the administration of prophylactic antibiotics. Therefore, as a next hierarchical level with higher resolution, we considered a group effect of prophylactic antibiotics (β*_o_*) from which each individual prophylactic antibiotic (fluoroquinolones, i.v. vancomycin, trimethoprim-sulfamethoxazole, and atovaquone) could deviate (1 | *O*, partial pooling of the effects of antibiotic prophylaxis). Finally, and with highest resolution, our model included independent variables for empirical antibiotics: piperacillin-tazobactam, meropenem, metronidazole, cephalosporins (generations 1 to 3), vancomycin (p.o.), cefepime, and linezolid were considered without pooling.

We accounted for repeated samples from the same patient by including a random intercept term (1 | *P*). Finally, we included a term that limits the otherwise exponential growth of anaerobes at high densities (the capacity term of the logistic growth equation, with associated parameter β*c*). Changes in the anaerobe abundance (*N*) were modeled as:

$$\frac{\text{log}\left(N_{t}\right)-\text{log}(N_{t-\text{Δ}t})}{\text{Δ}t}=N(r+{\text{β}}_{p1}\text{ phase I}+{\text{β}}_{p2}\text{ phase II}+\underset{i\in I}{\sum }{\text{β}}_{\text{ai}}A_{i}+{\text{β}}_{o}+\left(1|O\right)+\left(1|P\right)+{\text{β}}_{c}N_{t},\sigma_{m})$$

i.e., as a normally (𝒩) distributed variable that is a function of intrinsic growth rate (*r*), the effects of phase I (β*_p_*_1_) and phase II (β*_p_*_2_) and the growth rate-changing effects, β_ai_, of empirical antibiotics (*A_i_*) and antibiotic prophylaxis (β*_o_*, *O*), with the residual uncaptured variance of the model, σ*_m_*). As for phase I and II, we constructed binary antibiotic covariate indicators (1 if an antibiotic was administered during the interval [*t*, *t* − Δ*t*]; 0 otherwise).

We used uninformative priors [𝒩(0,100^2^)] for the growth rate, empirical antibiotics, and the HCT treatment phases and regularizing priors [𝒩(0, 10^−1^)] for the other parameters. This analysis produced posterior distributions for each parameter after “no U-turn” sampling of 10,000 samples from 3 traces (34), each corresponding to an estimate of the degree of impact on obligate anaerobic bacterial populations.

We used the posterior parameter distributions to assess our model. We simulated the predicted changes for each patient’s timeline, starting with the first observed anaerobe count from that patient. We sampled 100 posterior predictions of anaerobe changes between time points and used the mean predicted change for the calculation of the anaerobe count in the next time step.

To describe the effect of realistic antibiotic treatment regimens on the group of commensal anaerobes in HCT patients, we compiled a list of all antibiotic administration courses as they occurred in our patient group, i.e., the duration of administration, the period when the antibiotic was administered (e.g., phase I or phase II), and other coadministered antibiotics. We then repeatedly chose a random antibiotic course from this list, with replacement, and assigned parameters chosen jointly from the posterior parameter value distributions to our model. Then, starting from an initial, normalized density set to 1, we used the model to calculate the predicted fold change of anaerobe density at the end of each antibiotic course. Aggregating all of these fold change values allowed us to calculate the average residual fraction of anaerobes after a typical course of specific antibiotics.

## Supplementary Material

Supplemental file 1

Supplemental file 2

Supplemental file 3

Supplemental file 4

Supplemental file 5

Supplemental file 6

Supplemental file 7

Supplemental file 8
