# Supplementary figures and images for: Antibiotic-Induced Shifts in Fecal Microbiota Density and Composition during Hematopoietic Stem Cell Transplantation
Source: Infect Immun. 2019 Aug 21;87(9):e00206-19. doi: 10.1128/IAI.00206-19 (PMC6704593; doi:10.1128/IAI.00206-19)

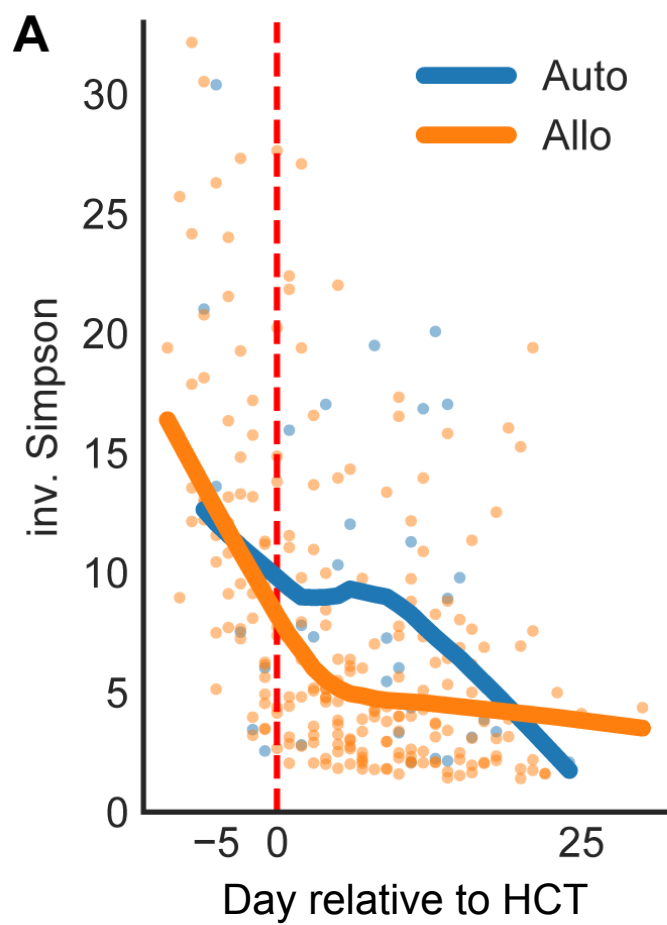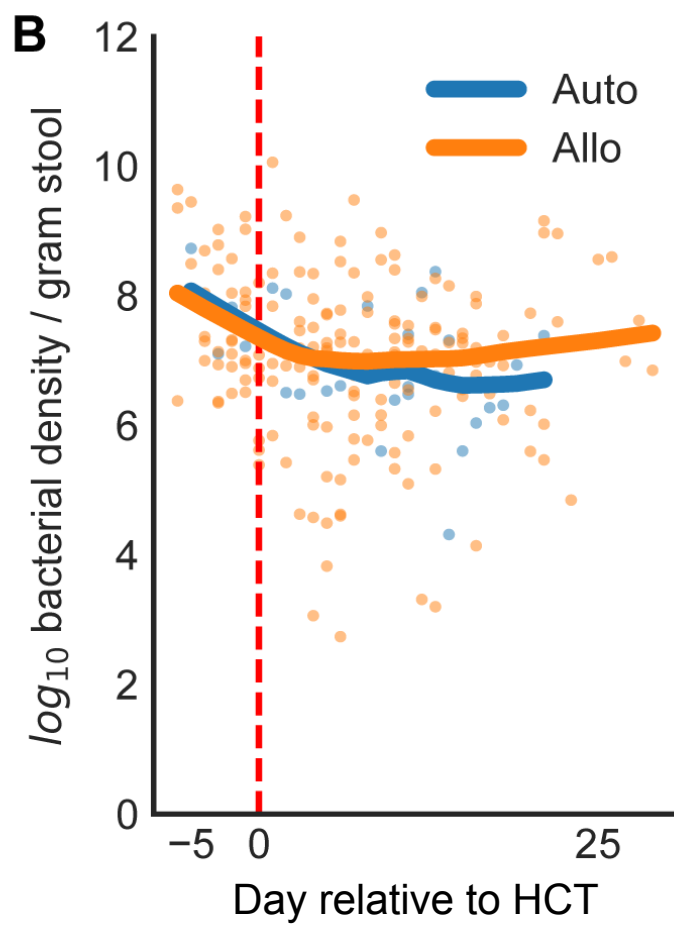

Supplement: Supplemental file 1 [file IAI.00206-19-s0001.pdf]

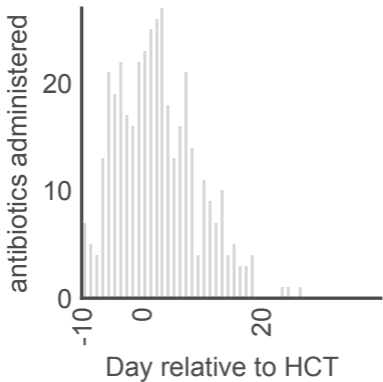

Supplement: Supplemental file 2 [file IAI.00206-19-s0002.pdf]

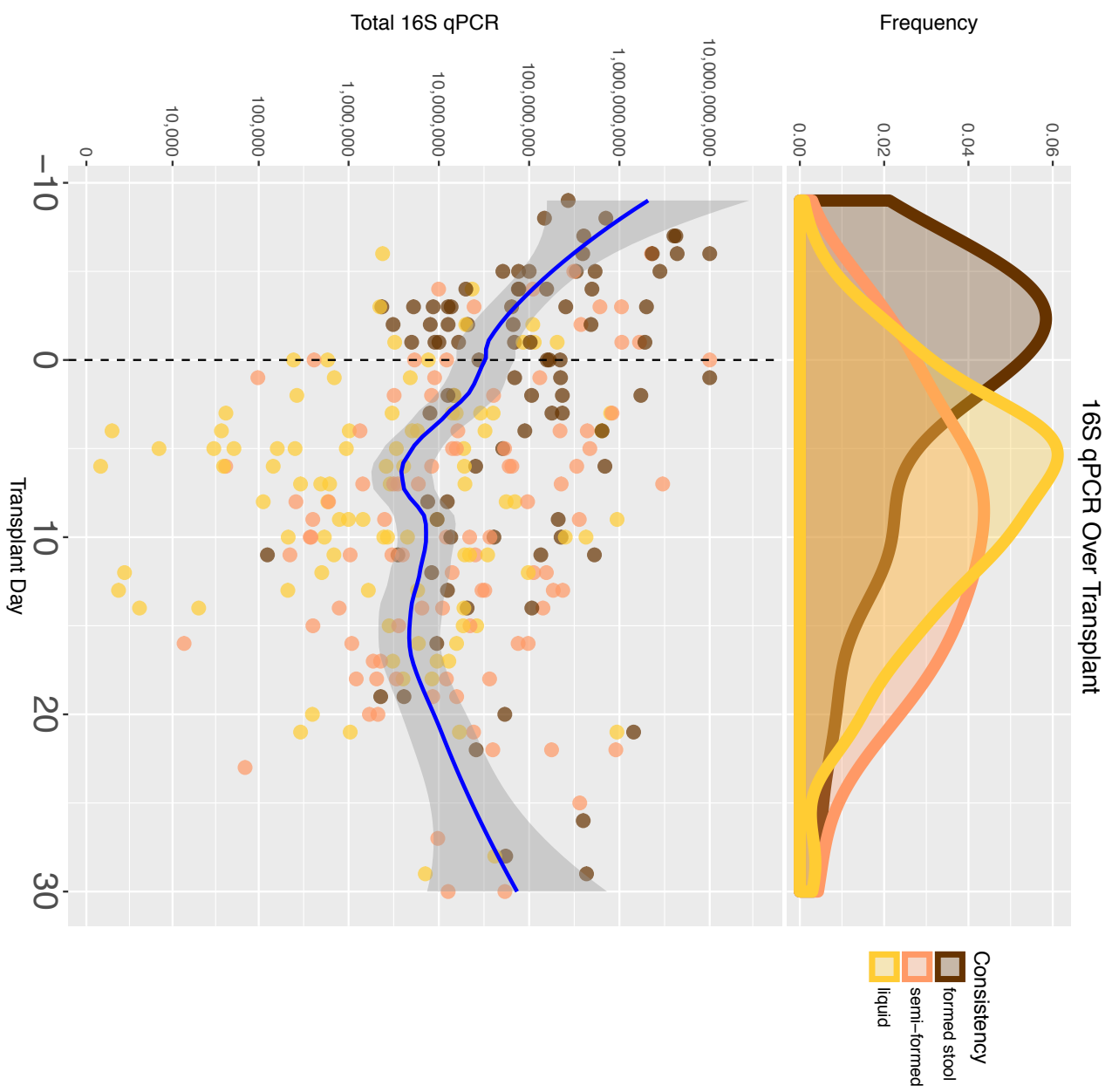

Supplement: Supplemental file 3 [file IAI.00206-19-s0003.pdf]

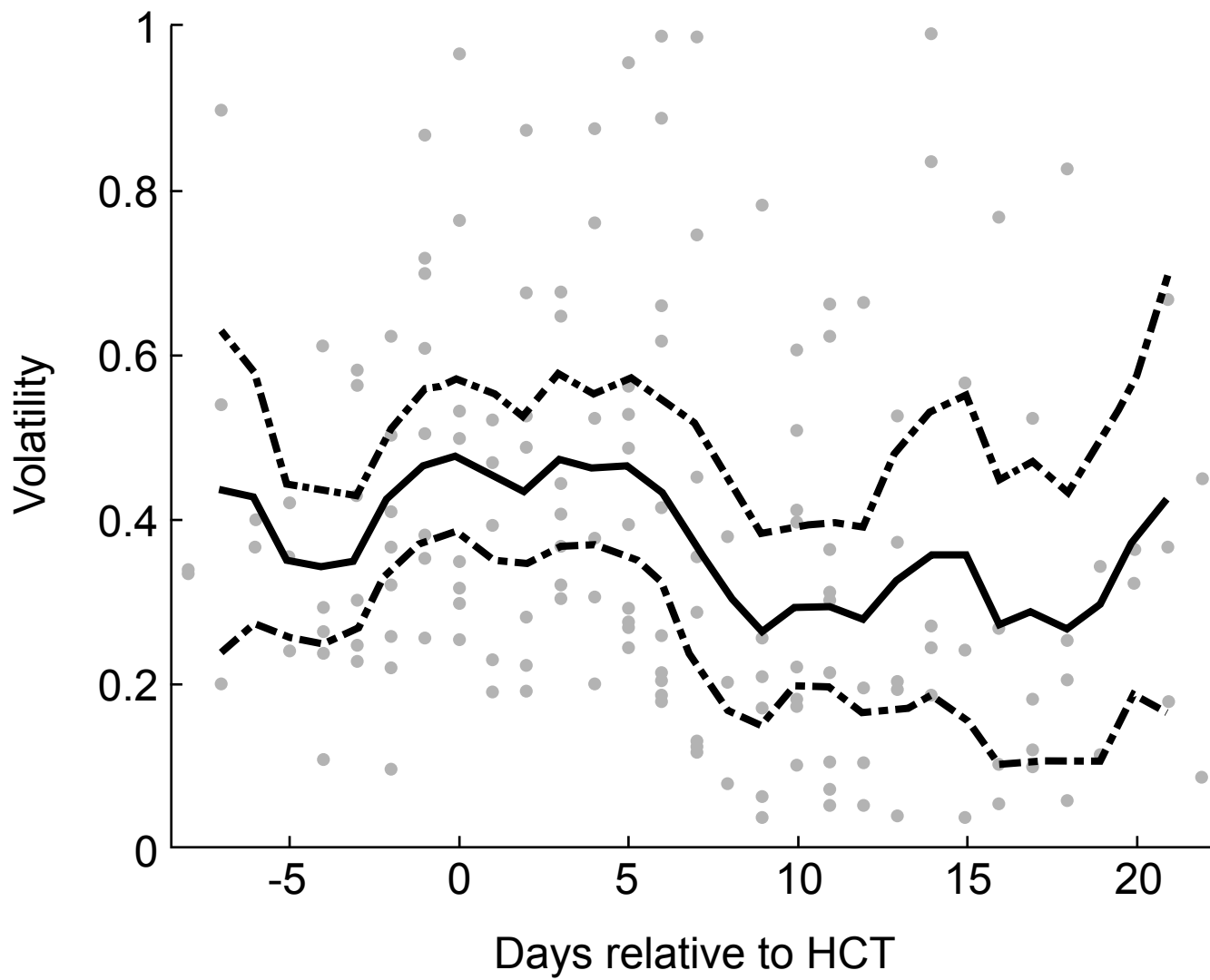

Supplement: Supplemental file 5 [file IAI.00206-19-s0005.pdf]

# Anaerobe timecourses

log-Anaerobes counts / gram of stool

Observed  
Predicted

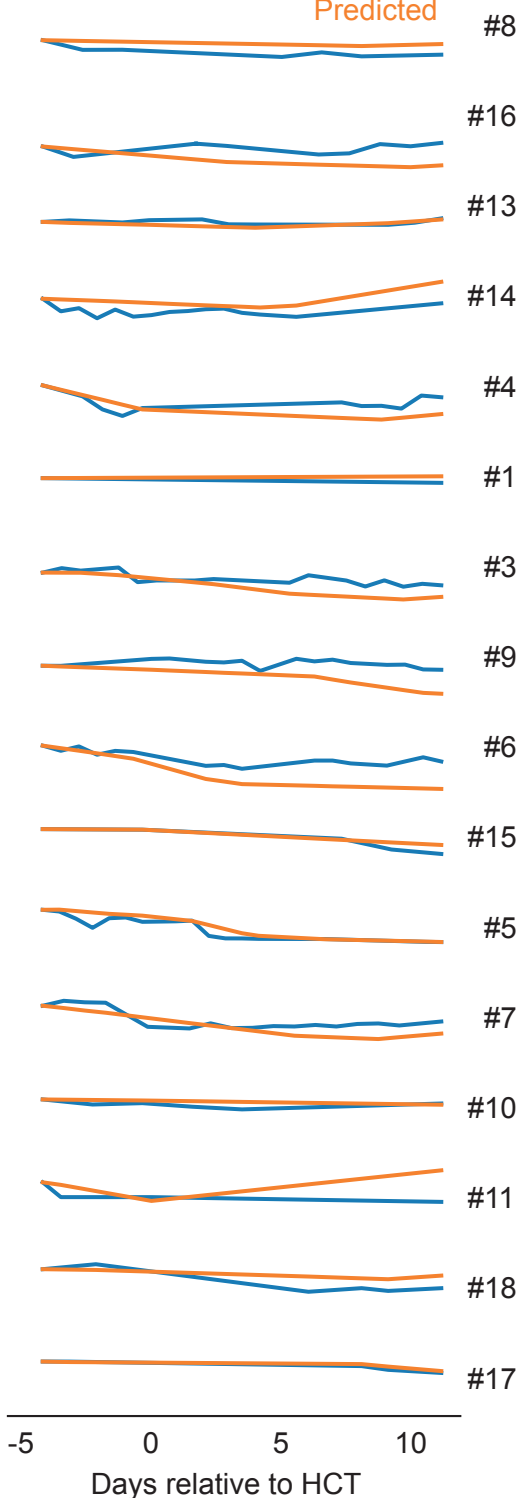

Supplement: Supplemental file 6 [file IAI.00206-19-s0006.pdf]
