# Supplementary material for: Antibiotic-Induced Shifts in Fecal Microbiota Density and Composition during Hematopoietic Stem Cell Transplantation
Source: Infect Immun. 2019 Aug 21;87(9):e00206-19. doi: 10.1128/IAI.00206-19 (PMC6704593; doi:10.1128/IAI.00206-19)
Supplement: Supplemental file 4 [file IAI.00206-19-s0004.pdf]

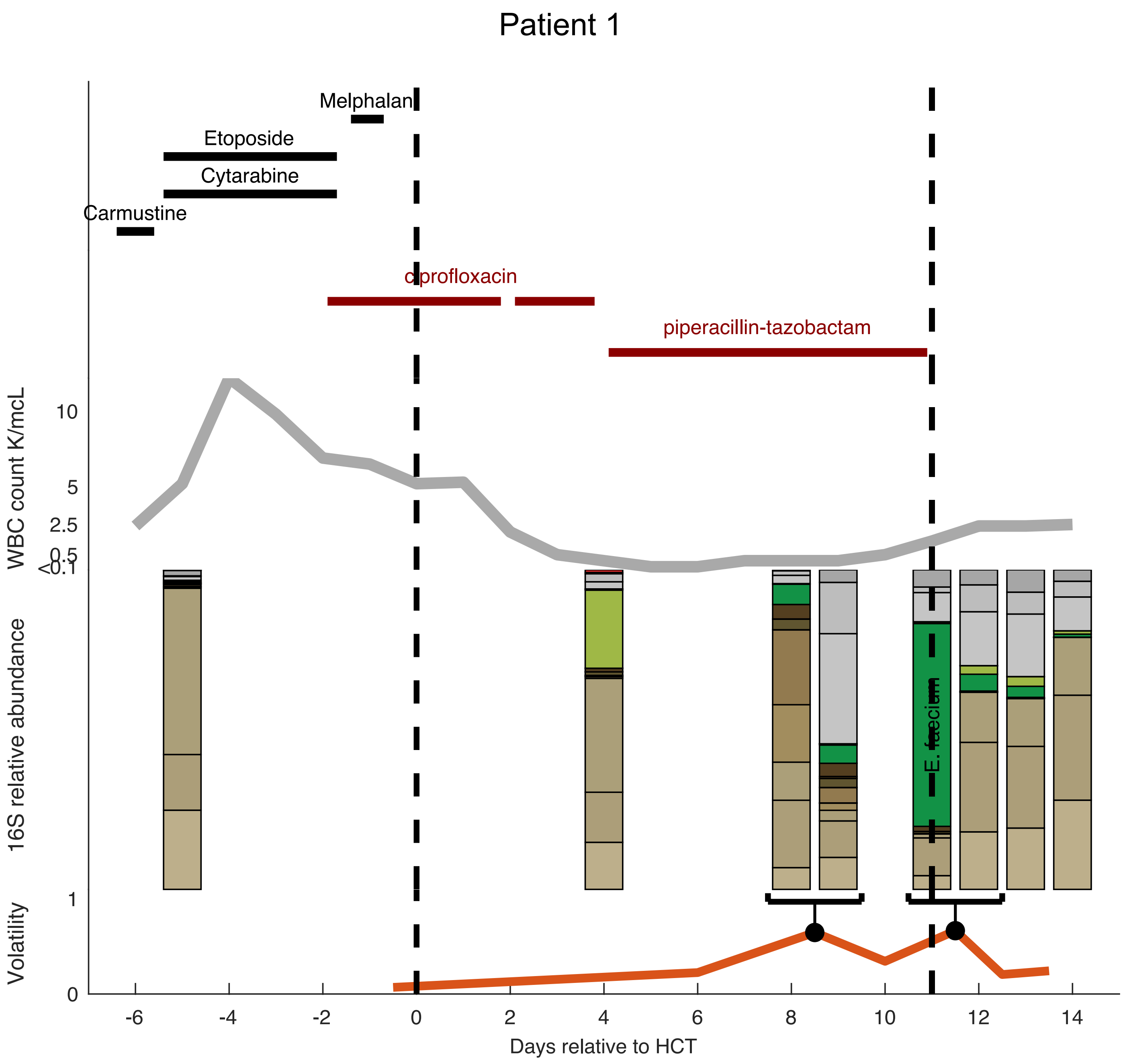

Patient 1
Melphalan
Etoposide
Cytarabine
Carmustine
ciprofloxacin
piperacillin-tazobactam
WBC count K/mcL
10
5
2.5
0.5
<0.1
16S relative abundance
E. faecium
Volatility
1
0
-6
-4
-2
0
2
4
6
8
10
12
14
Days relative to HCT

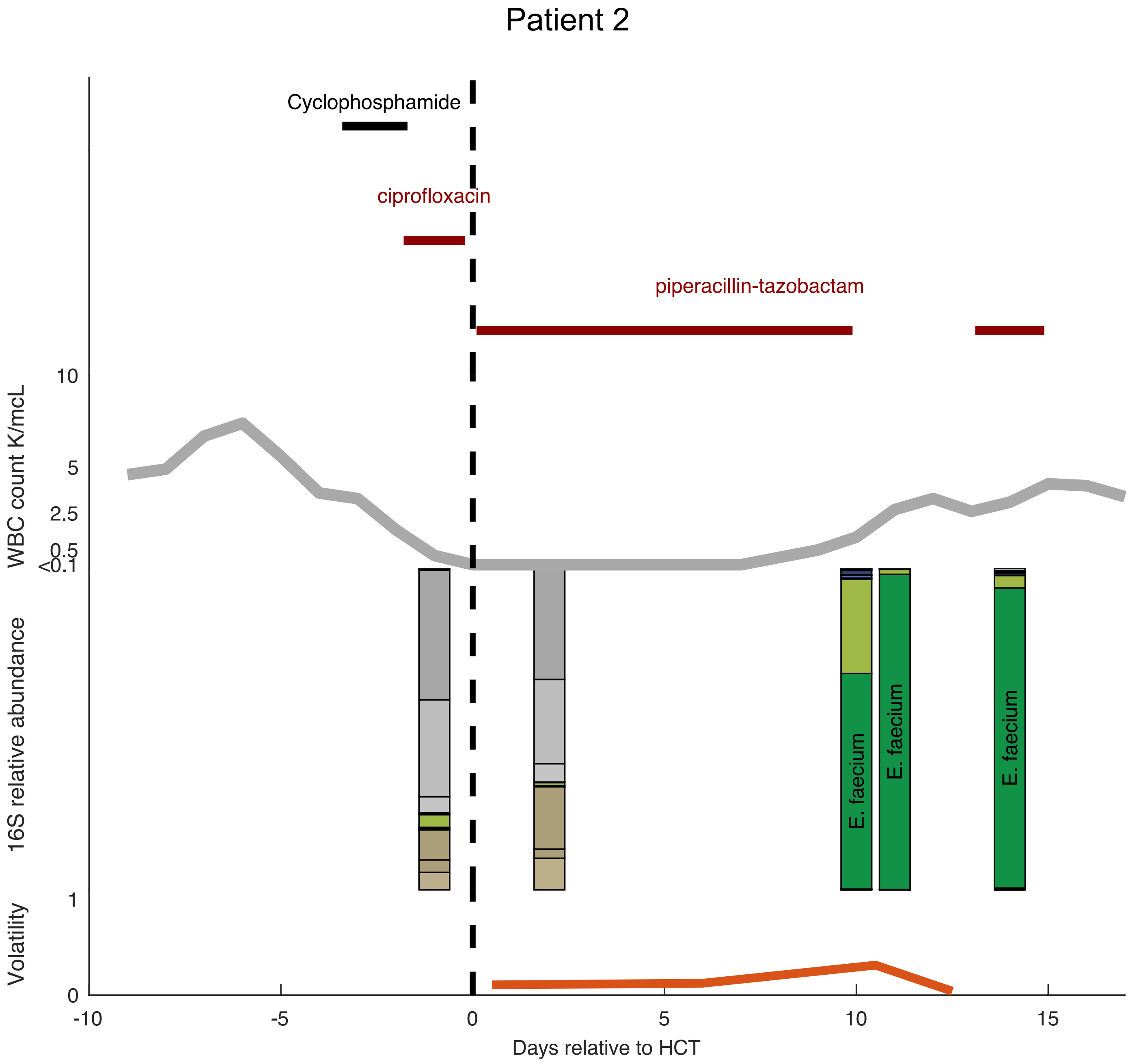

Patient 2
Cyclophosphamide
ciprofloxacin
piperacillin-tazobactam
WBC count K/mcL
10
5
2.5
0.5
<0.1
16S relative abundance
E. faecium
E. faecium
E. faecium
Volatility
1
0
-10
-5
0
5
10
15
Days relative to HCT

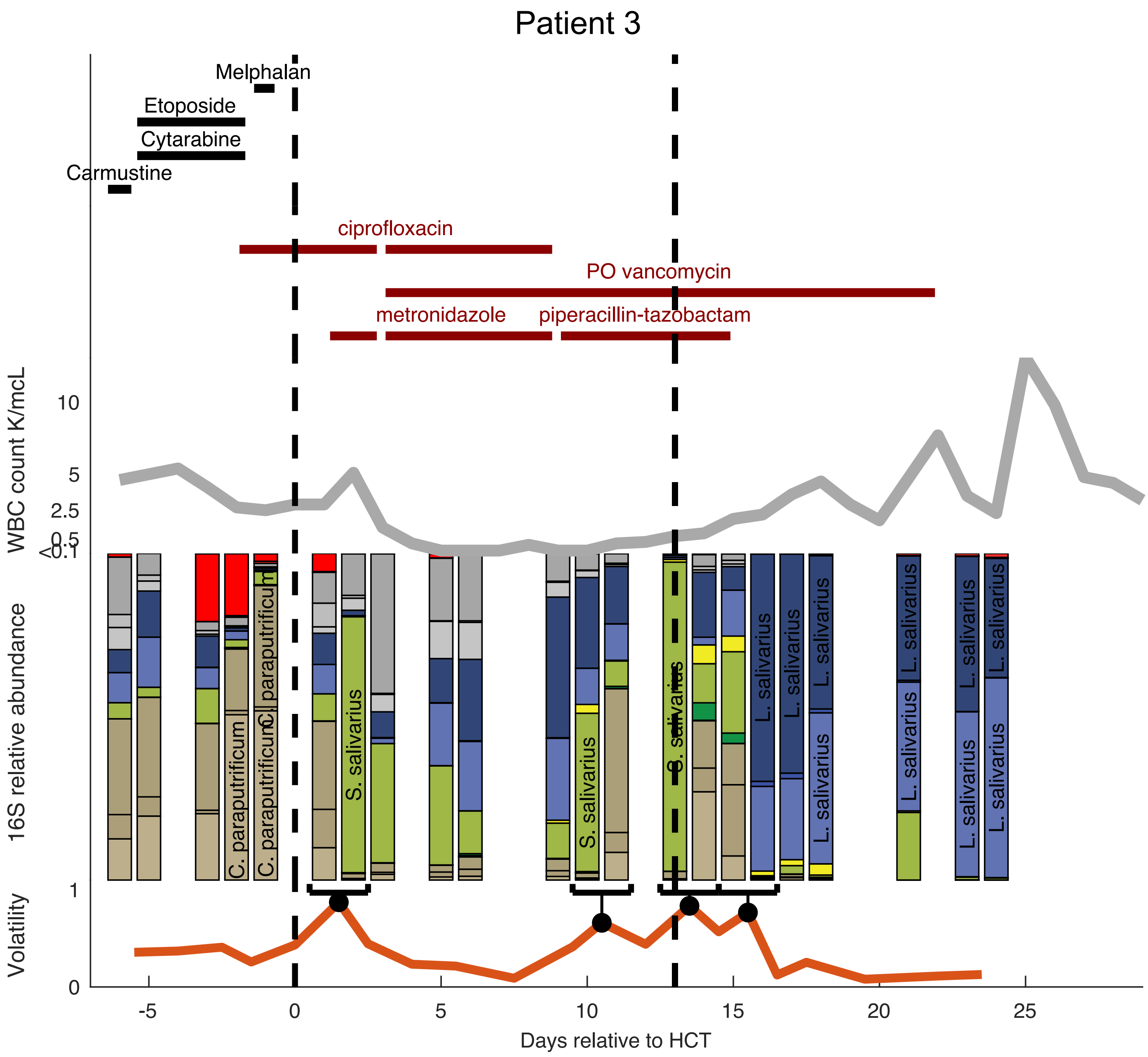

Patient 3
Melphalan
Etoposide
Cytarabine
Carmustine
ciprofloxacin
PO vancomycin
metronidazole
piperacillin-tazobactam
WBC count K/mcL
10
5
2.5
0.5
<0.1
16S relative abundance
C. paraputrificum
C. paraputrificum
C. paraputrificum
S. salivarius
S. salivarius
S. salivarius
L. salivarius
L. salivarius
L. salivarius
L. salivarius
L. salivarius
L. salivarius
L. salivarius
L. salivarius
L. salivarius
L. salivarius
L. salivarius
Volatility
1
0
-5
0
5
10
15
20
25
Days relative to HCT

# Patient 15

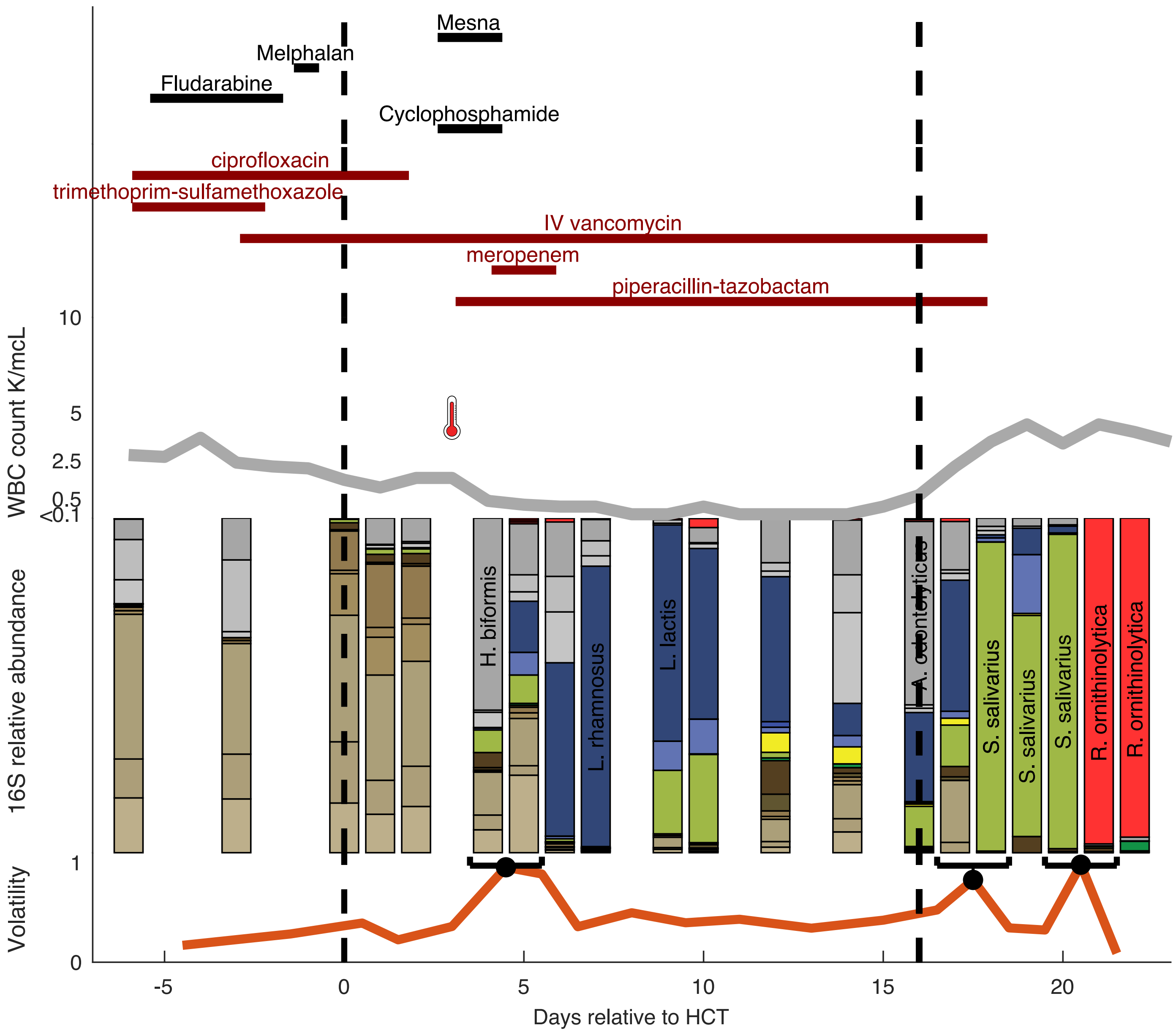

Mesna
Melphalan
Fludarabine
Cyclophosphamide
ciprofloxacin
trimethoprim-sulfamethoxazole
IV vancomycin
meropenem
piperacillin-tazobactam
WBC count K/mcL
10
5
2.5
0.5
<0.1
16S relative abundance
H. biformis
L. rhamnosus
L. lactis
A. odontolyticus
S. salivarius
S. salivarius
S. salivarius
R. ornithinolytica
R. ornithinolytica
Volatility
1
0
-5
0
5
10
15
20
Days relative to HCT

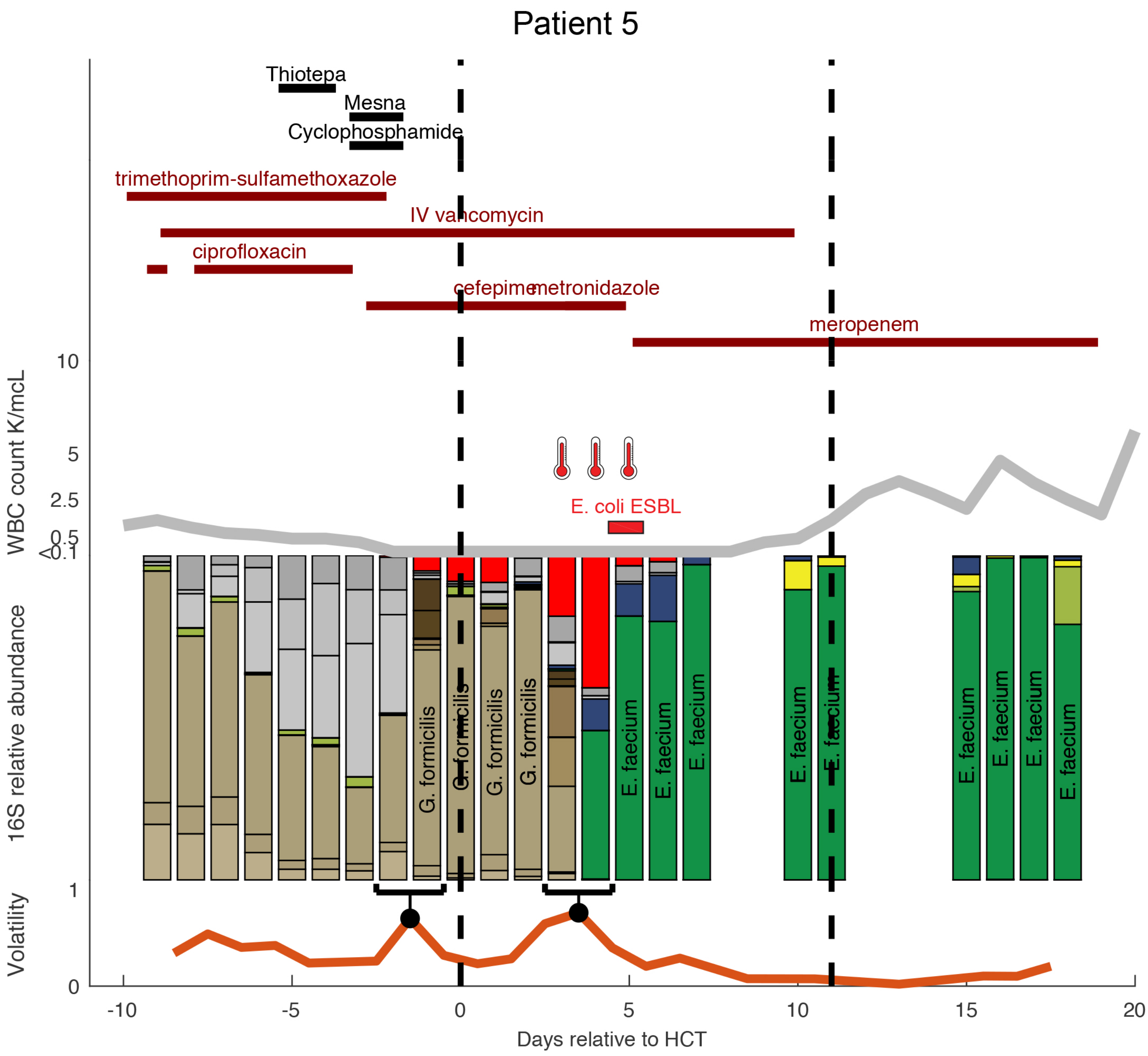

Patient 5
Thiotepa
Mesna
Cyclophosphamide
trimethoprim-sulfamethoxazole
IV vancomycin
ciprofloxacin
cefepime
metronidazole
meropenem
E. coli ESBL
WBC count K/mcL
10
5
2.5
0.5
<0.1
16S relative abundance
G. formicilis
E. faecium
Volatility
1
0
-10
-5
0
5
10
15
20
Days relative to HCT

Patient 6

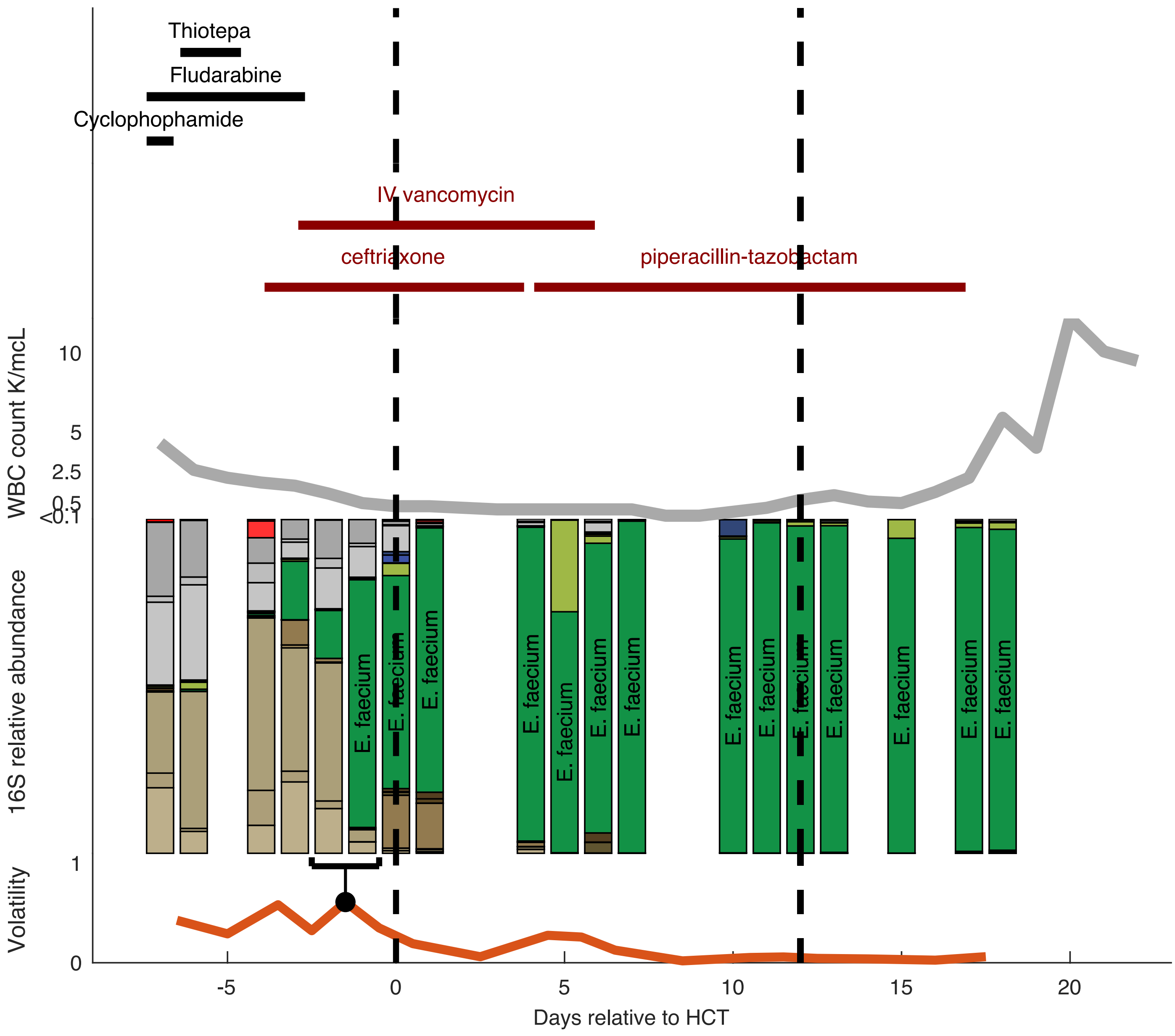

Thiotepa
Fludarabine
Cyclophophamide
IV vancomycin
ceftriaxone
piperacillin-tazobactam
WBC count K/mcL
10
5
2.5
0.5
<0.1
16S relative abundance
E. faecium
1
Volatility
0
-5
0
5
10
15
20
Days relative to HCT

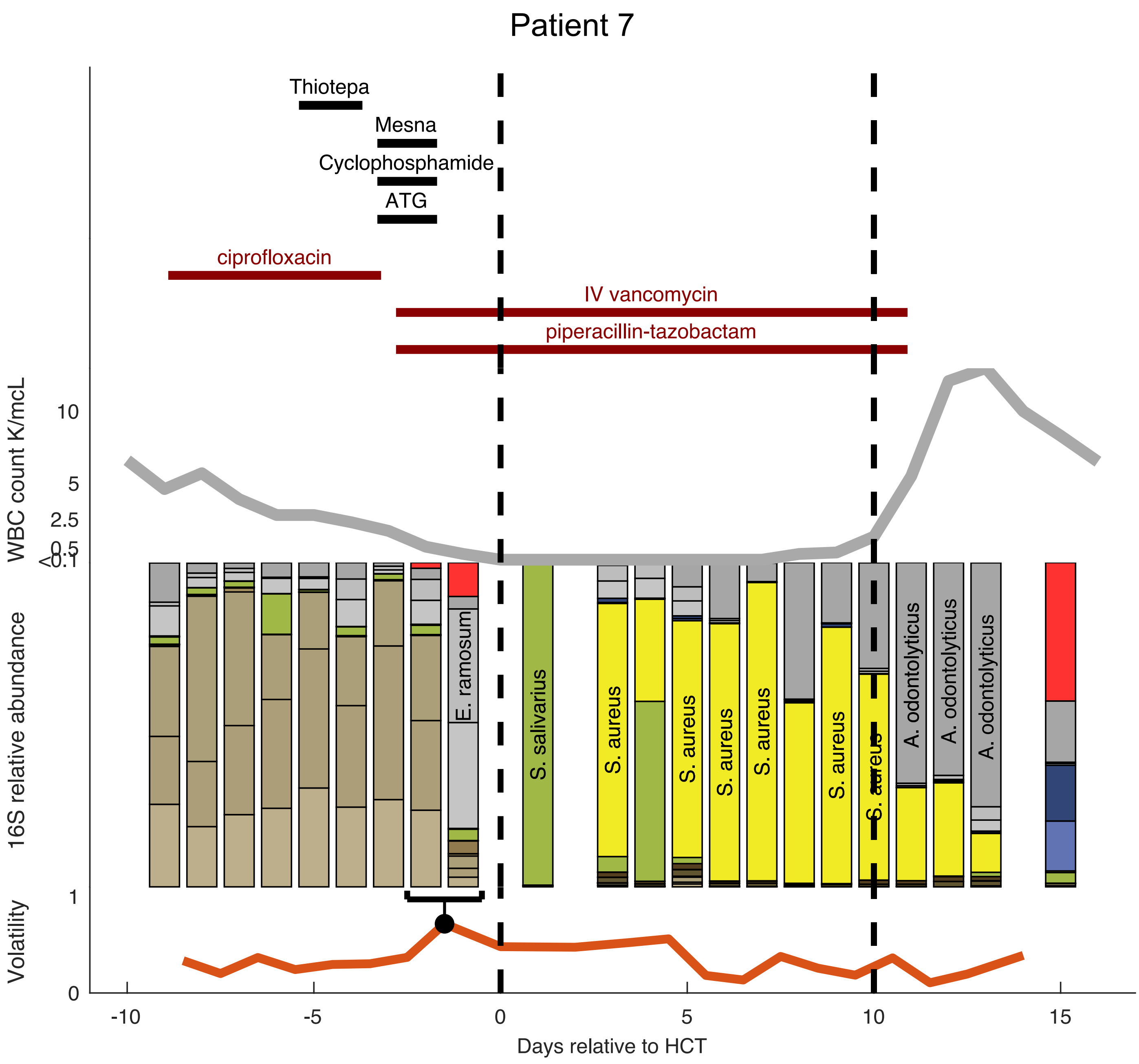

Patient 7
Thiotepa
Mesna
Cyclophosphamide
ATG
ciprofloxacin
IV vancomycin
piperacillin-tazobactam
WBC count K/mcL
10
5
2.5
0.5
<0.1
16S relative abundance
E. ramosum
S. salivarius
S. aureus
S. aureus
S. aureus
S. aureus
S. aureus
S. aureus
A. odontolyticus
A. odontolyticus
A. odontolyticus
Volatility
1
0
-10
-5
0
5
10
15
Days relative to HCT

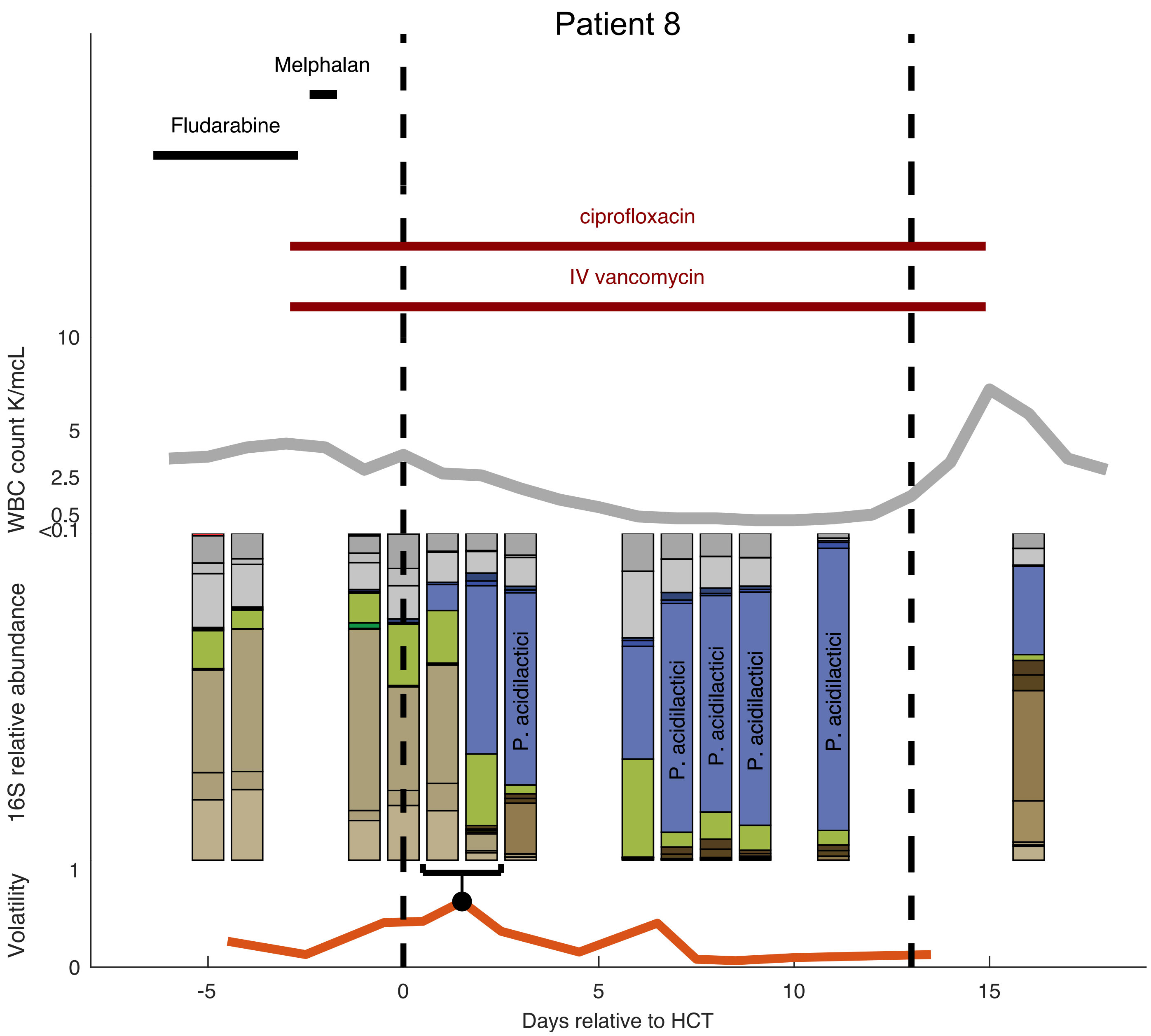

Patient 8
Melphalan
Fludarabine
ciprofloxacin
IV vancomycin
WBC count K/mcL
10
5
2.5
0.5
<0.1
16S relative abundance
P. acidilactici
P. acidilactici
P. acidilactici
P. acidilactici
P. acidilactici
Volatility
1
0
-5
0
5
10
15
Days relative to HCT

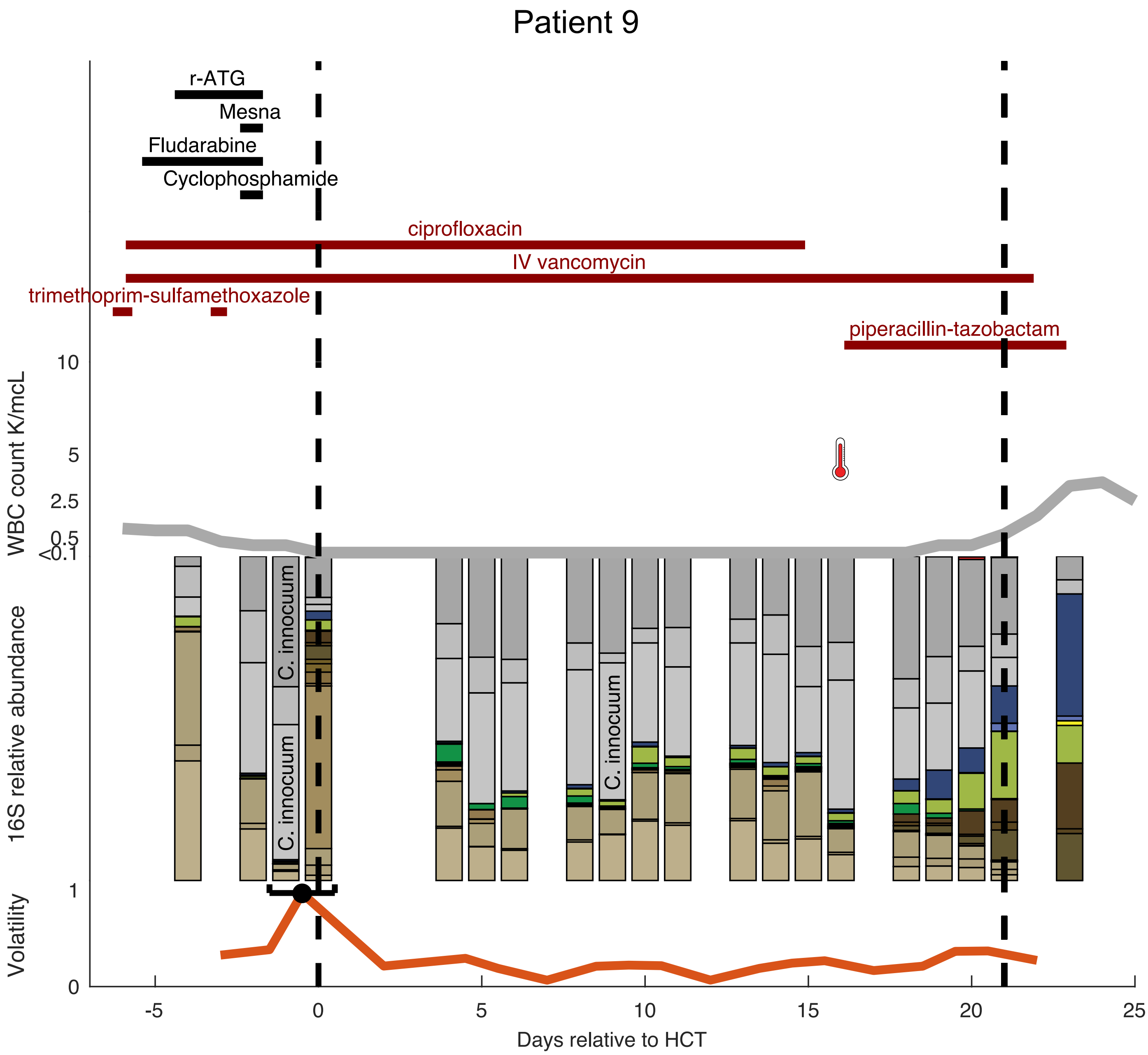
Patient 9
r-ATG
Mesna
Fludarabine
Cyclophosphamide
ciprofloxacin
IV vancomycin
trimethoprim-sulfamethoxazole
piperacillin-tazobactam
WBC count K/mcL
10
5
2.5
0.5
<0.1
16S relative abundance
C. innocuum
C. innocuum
C. innocuum
Volatility
1
0
-5
0
5
10
15
20
25
Days relative to HCT

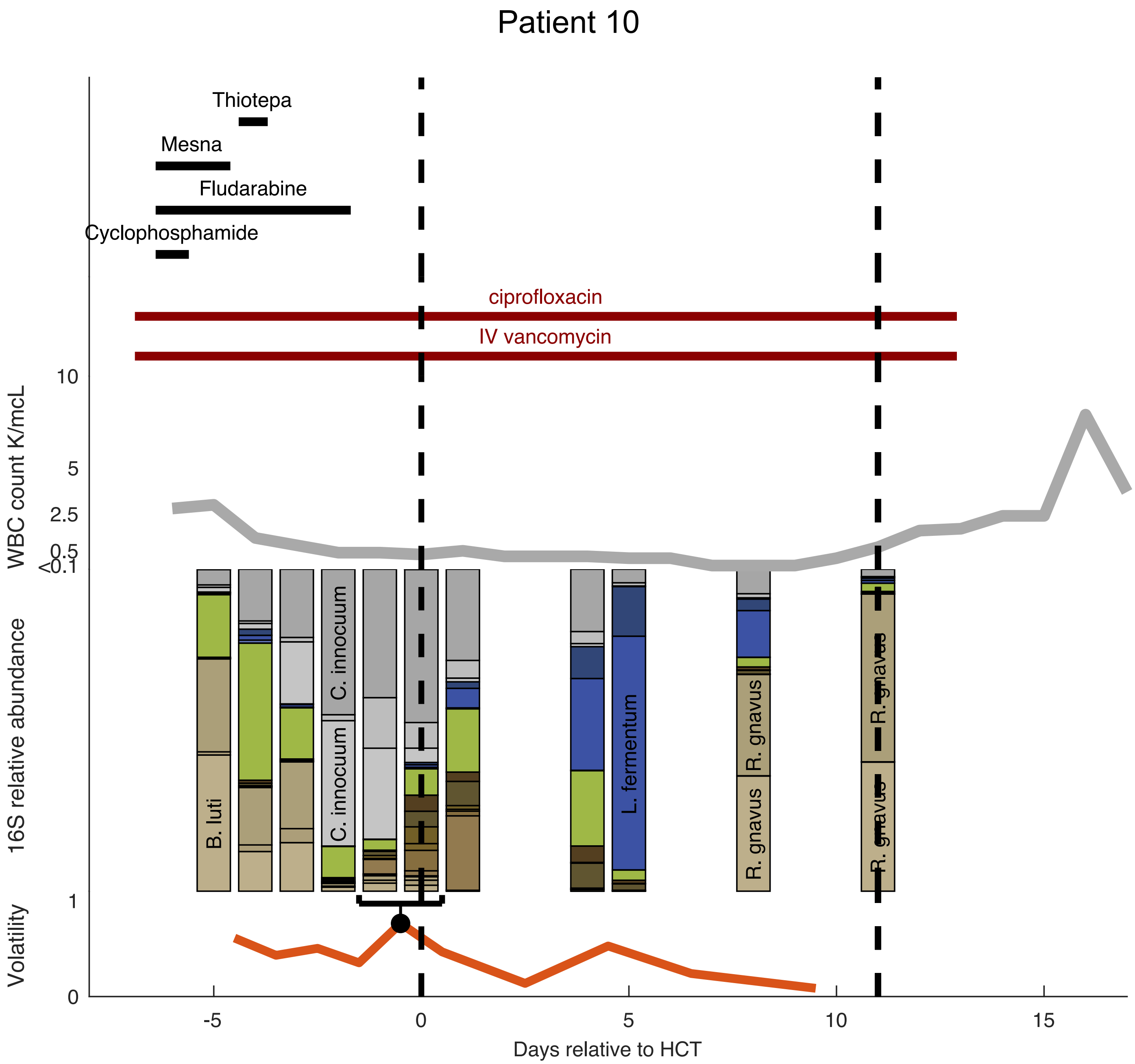

Patient 10
Thiotepa
Mesna
Fludarabine
Cyclophosphamide
ciprofloxacin
IV vancomycin
10
5
2.5
0.5
<0.1
WBC count K/mcL
16S relative abundance
1
0
Volatility
B. luti
C. innocuum
C. innocuum
L. fermentum
R. gnavus
R. gnavus
R. gnavus
R. gnavus
-5
0
5
10
15
Days relative to HCT

# Patient 11

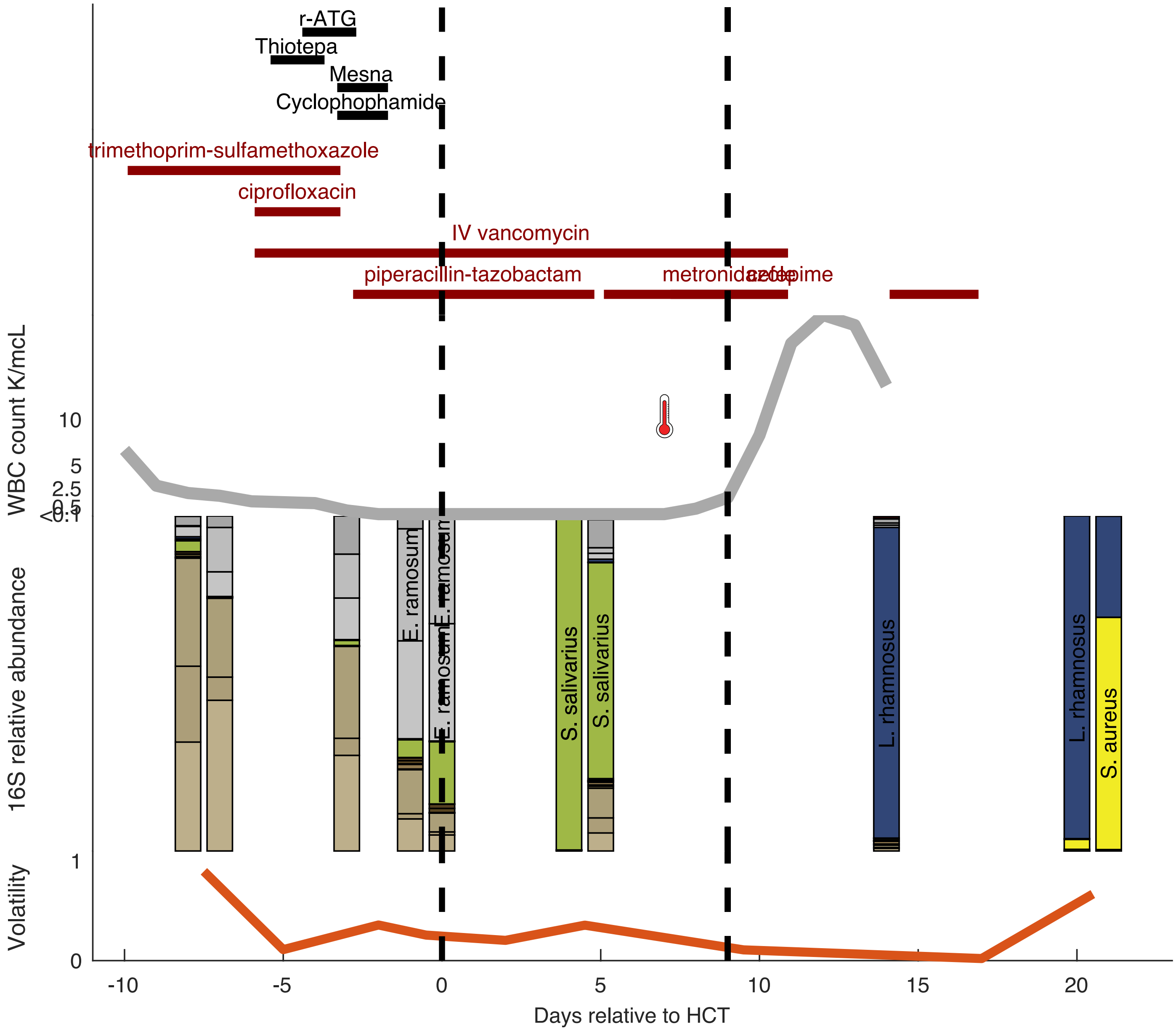

r-ATG
Thiotepa
Mesna
Cyclophophamide
trimethoprim-sulfamethoxazole
ciprofloxacin
IV vancomycin
piperacillin-tazobactam
metronidazole
cefepime
WBC count K/mcL
10
5
2.5
0.5
<0.1
16S relative abundance
E. ramosum
E. ramosum
S. salivarius
S. salivarius
L. rhamnosus
L. rhamnosus
S. aureus
Volatility
1
0
-10
-5
0
5
10
15
20
Days relative to HCT

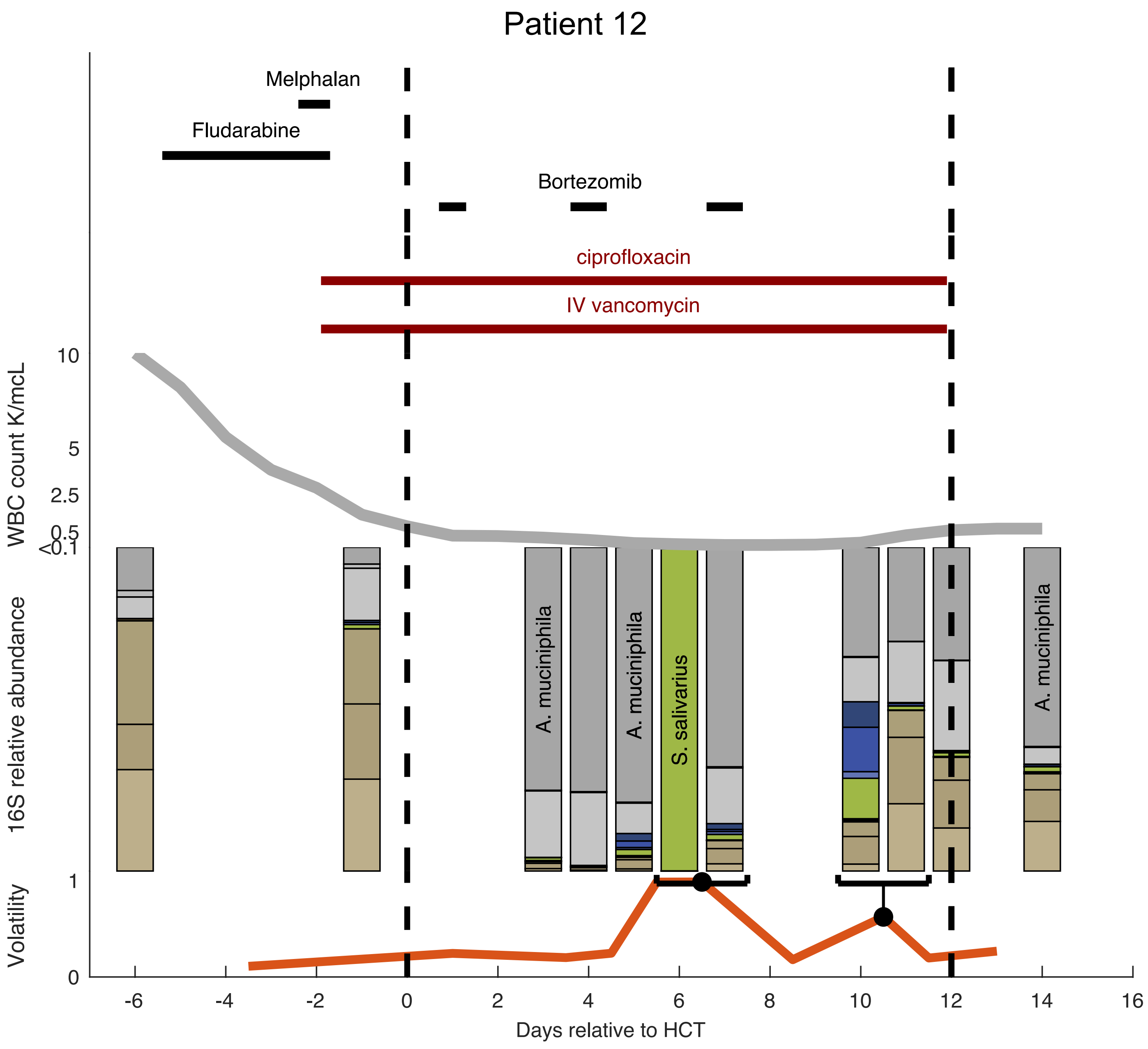

Patient 12
Melphalan
Fludarabine
Bortezomib
ciprofloxacin
IV vancomycin
WBC count K/mcL
10
5
2.5
0.5
<0.1
16S relative abundance
A. muciniphila
A. muciniphila
S. salivarius
A. muciniphila
Volatility
1
0
-6
-4
-2
0
2
4
6
8
10
12
14
16
Days relative to HCT

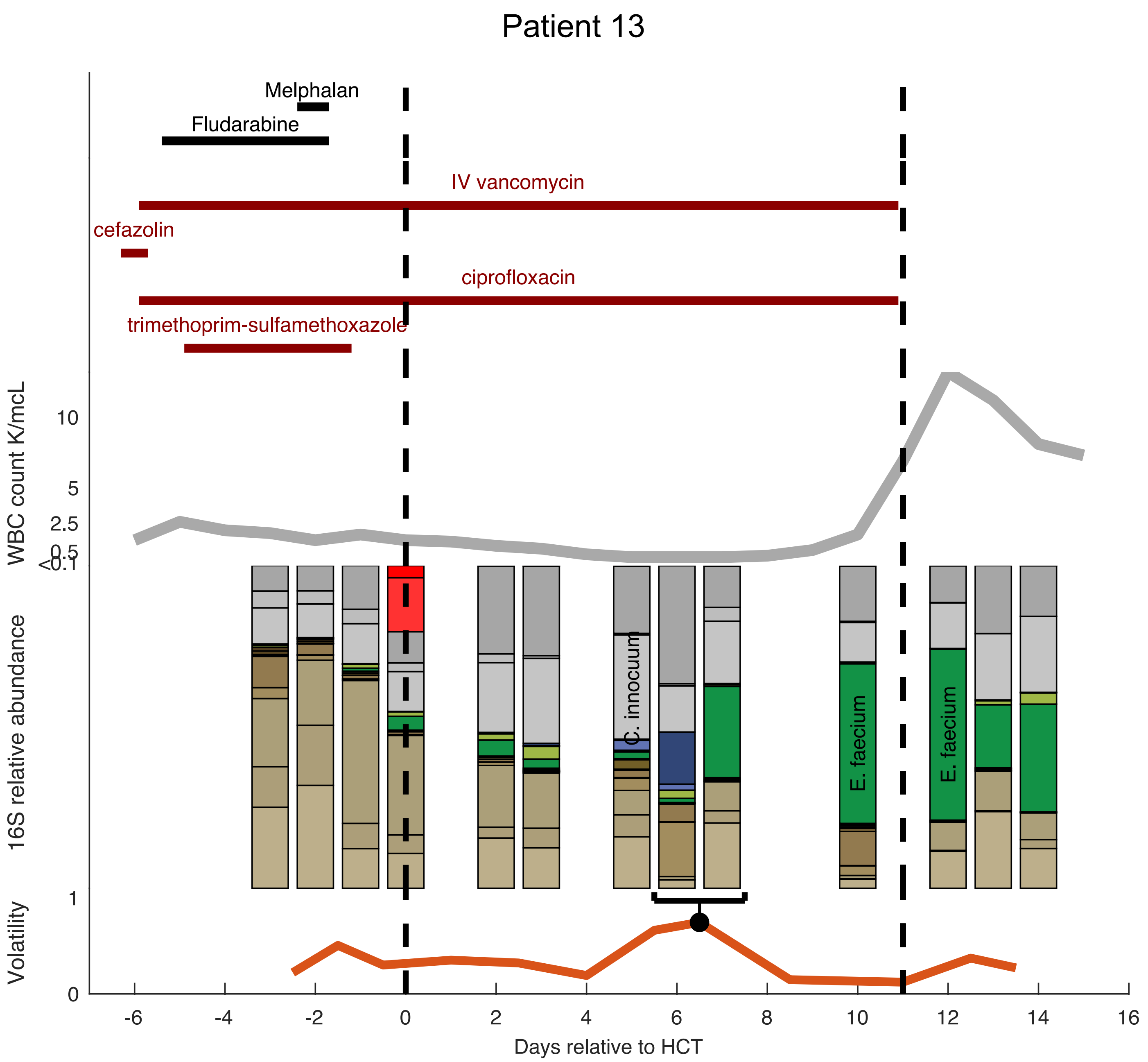
Patient 13
Melphalan
Fludarabine
IV vancomycin
cefazolin
ciprofloxacin
trimethoprim-sulfamethoxazole
WBC count K/mcL
10
5
2.5
0.5
<0.1
16S relative abundance
C. innocuum
E. faecium
E. faecium
Volatility
1
0
-6
-4
-2
0
2
4
6
8
10
12
14
16
Days relative to HCT

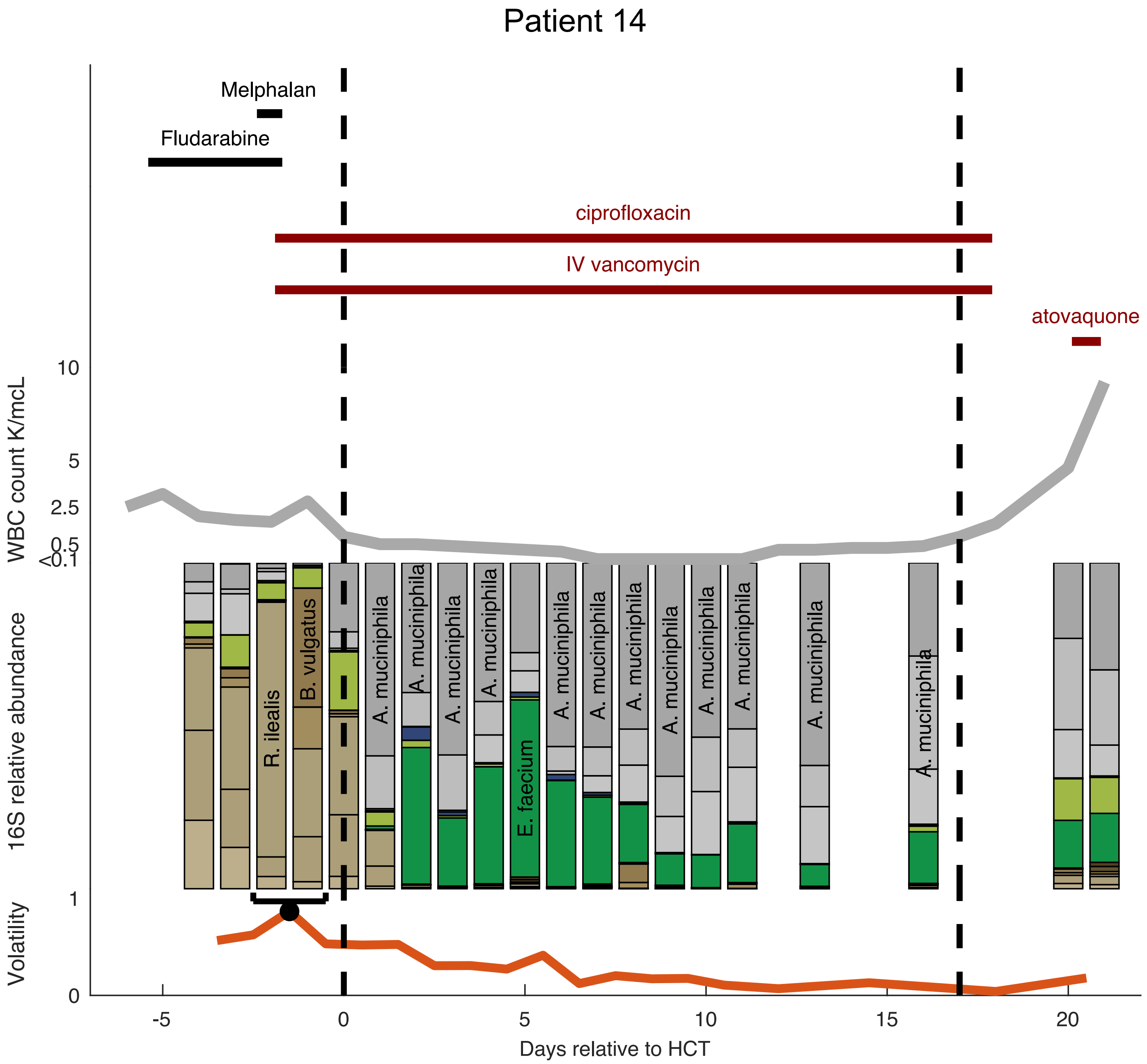
Patient 14
Melphalan
Fludarabine
ciprofloxacin
IV vancomycin
atovaquone
WBC count K/mcL
10
5
2.5
0.5
<0.1
16S relative abundance
R. ilealis
B. vulgatus
A. muciniphila
E. faecium
Volatility
1
0
-5
0
5
10
15
20
Days relative to HCT

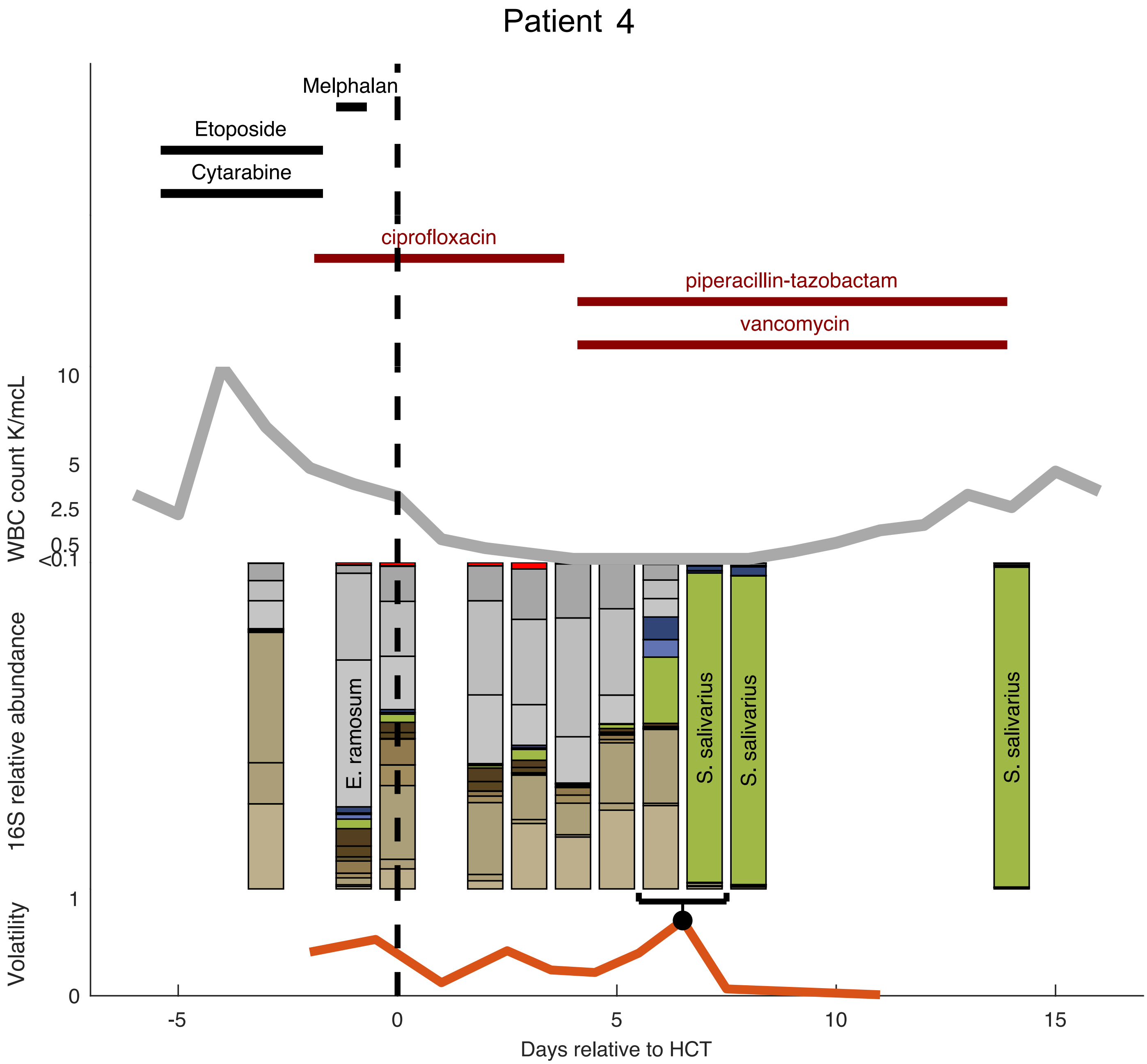

Patient 4
Melphalan
Etoposide
Cytarabine
ciprofloxacin
piperacillin-tazobactam
vancomycin
WBC count K/mcL
10
5
2.5
0.5
<0.1
16S relative abundance
E. ramosum
S. salivarius
S. salivarius
S. salivarius
Volatility
1
0
-5
0
5
10
15
Days relative to HCT

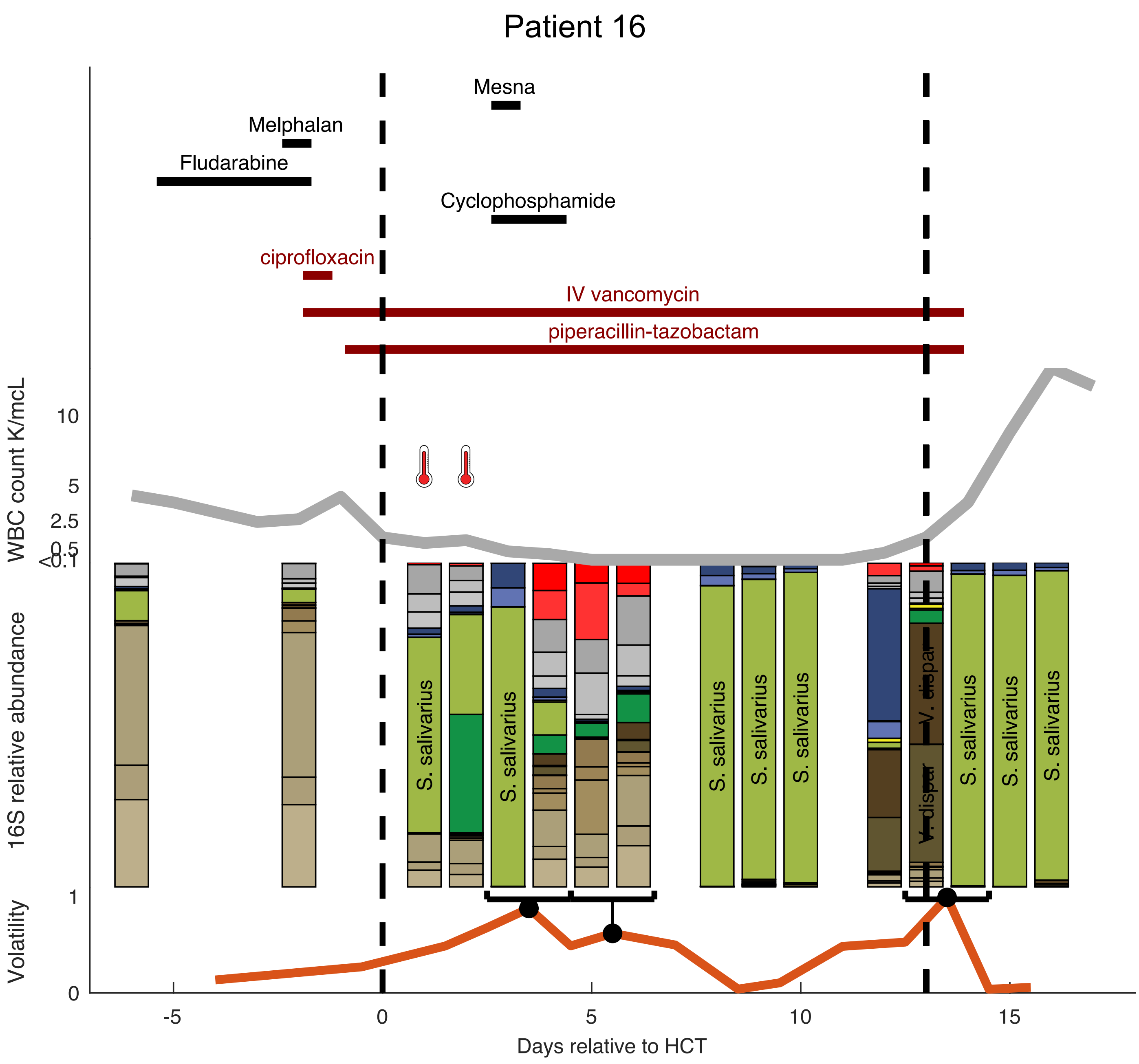

Patient 16
Mesna
Melphalan
Fludarabine
Cyclophosphamide
ciprofloxacin
IV vancomycin
piperacillin-tazobactam
WBC count K/mcL
10
5
2.5
0.5
<0.1
16S relative abundance
S. salivarius
S. salivarius
S. salivarius
S. salivarius
S. salivarius
S. salivarius
V. dispar
V. dispar
S. salivarius
S. salivarius
S. salivarius
Volatility
1
0
-5
0
5
10
15
Days relative to HCT

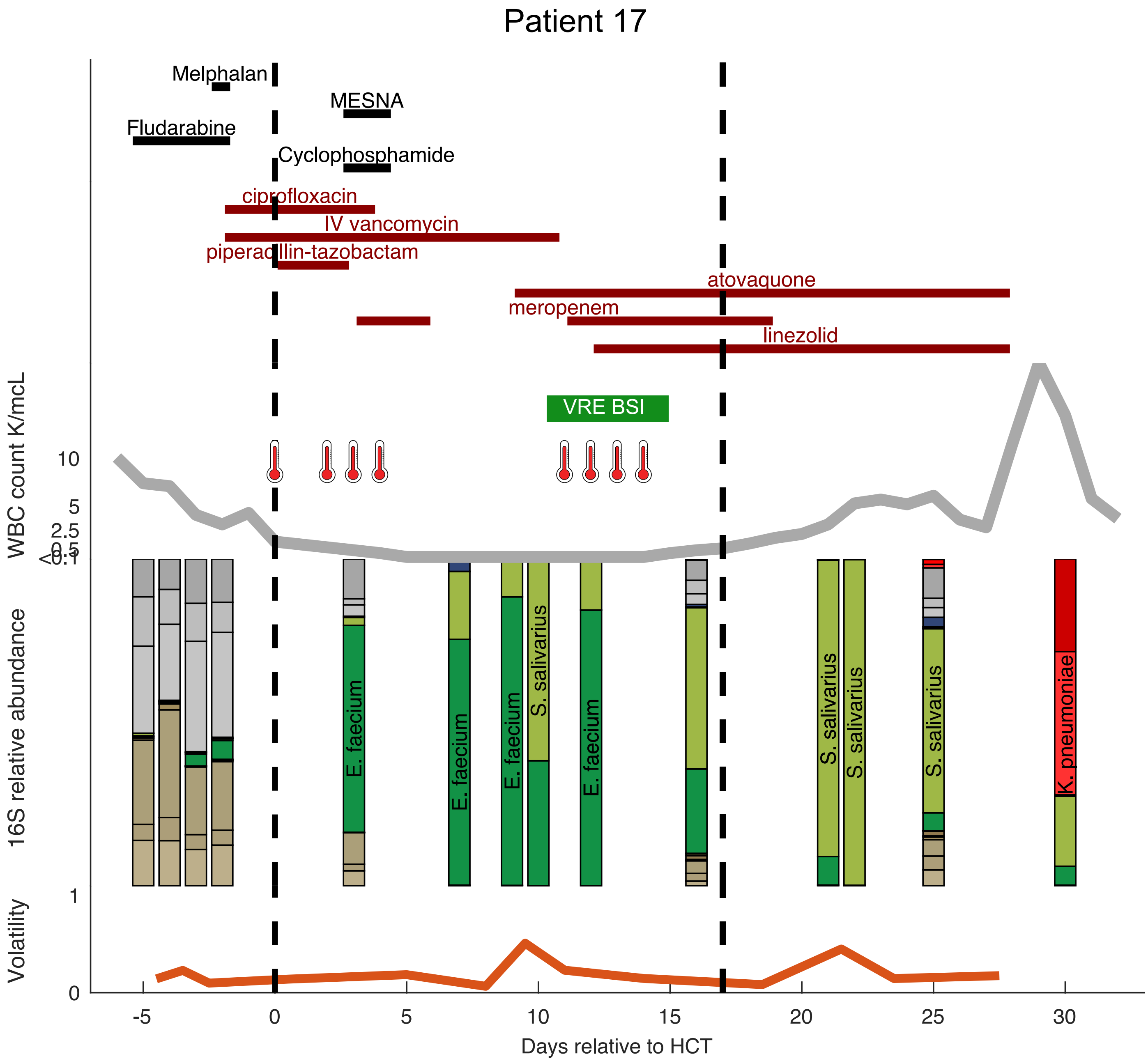

Patient 17
Melphalan
MESNA
Fludarabine
Cyclophosphamide
ciprofloxacin
IV vancomycin
piperacillin-tazobactam
atovaquone
meropenem
linezolid
VRE BSI
WBC count K/mcL
10
5
2.5
0.5
<0.1
16S relative abundance
E. faecium
E. faecium
E. faecium
S. salivarius
E. faecium
S. salivarius
S. salivarius
S. salivarius
K. pneumoniae
Volatility
1
0
-5
0
5
10
15
20
25
30
Days relative to HCT

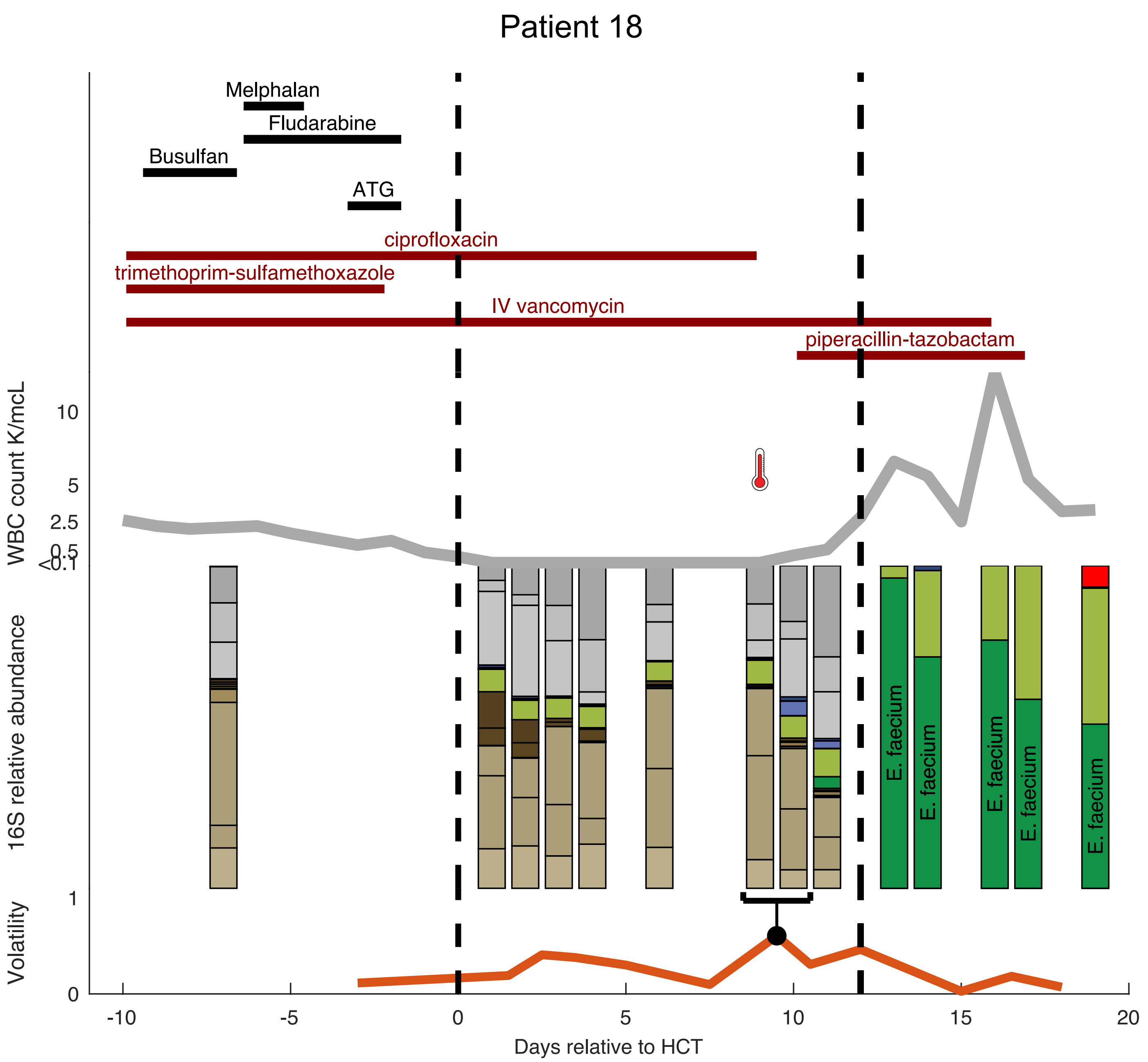
Patient 18
Melphalan
Fludarabine
Busulfan
ATG
ciprofloxacin
trimethoprim-sulfamethoxazole
IV vancomycin
piperacillin-tazobactam
WBC count K/mcL
10
5
2.5
0.5
<0.1
16S relative abundance
E. faecium
E. faecium
E. faecium
E. faecium
E. faecium
Volatility
1
0
-10
-5
0
5
10
15
20
Days relative to HCT
