# Supplementary material for: Antibiotic-Induced Shifts in Fecal Microbiota Density and Composition during Hematopoietic Stem Cell Transplantation
Source: Infect Immun. 2019 Aug 21;87(9):e00206-19. doi: 10.1128/IAI.00206-19 (PMC6704593; doi:10.1128/IAI.00206-19)
Supplement: Supplemental file 7 [file IAI.00206-19-s0007.pdf]

Supplementary table 1: MCMC parameter estimate results (mean estimate, HDI5, HDI95, R_hat) for antibiotics, HCT phases and growth rates (intercepts) from 3 independent MCMC runs with 10,000 samples each.

	**mean estimate**	**5% of all MCMC samples less than**	**95% of all MCMC samples less than**	**Gelman-Rubin statistic (R_hat)**
**Growth rate**	0.709	-0.128	1.555	1.001
**Phase I**	-0.627	-1.851	0.606	1.000
**Phase II**	-0.765	-1.683	0.119	1.000
**piperacillin tazobactam**	-0.928	-1.739	-0.073	1.000
**meropenem**	-1.015	-2.307	0.407	1.000
**metronidazole**	-0.505	-2.245	1.215	1.000
**cephalosporins (gen. 1-3)**	-0.850	-2.748	0.942	1.000
**vancomycin (PO)**	-0.352	-2.010	1.238	1.000
**cefepime**	-0.236	-1.852	1.437	1.000
**linezolid**	0.642	-3.843	4.847	1.000
**fluoroquinolones**	-0.081	-0.764	0.575	1.000
**vancomycin**	-0.242	-1.025	0.372	1.001
**trimethoprim-sulfamethoxazole**	-0.035	-0.864	0.817	1.000
**atovaquone**	0.383	-0.732	1.651	1.001
