# Supplementary material for: Antibiotic-Induced Shifts in Fecal Microbiota Density and Composition during Hematopoietic Stem Cell Transplantation
Source: Infect Immun. 2019 Aug 21;87(9):e00206-19. doi: 10.1128/IAI.00206-19 (PMC6704593; doi:10.1128/IAI.00206-19)
Supplement: Supplemental file 8 [file IAI.00206-19-s0008.pdf]

Supplementary Figure Captions:

Supplementary Figure 1: Microbiota destruction during HCT. During conditioning, before stem cell transfusion (day 0), the community diversity (A) of the microbiota in both allo- and auto-HCT patients tended to decline rapidly. Similarly, the bacterial density, measured as the total number of bacterial cells per gram of stool (B), also declined, with slight recovery of cell counts in allo-HCT patients during later days of hospitalization.

Supplementary Figure 2: Total counts of antibiotics prescribed per day relative to HCT, summed over all 18 patients.

Supplementary Figure 3: Relationship between total 16S copy number assessed by qPCR and stool consistency. The scatterplot shows the decline (and recovery) in total 16S copies per gram of stool wet weight over time (in days relative to HCT); line and shaded region: locally estimated scatterplot smoothing (LOESS) and confidence intervals. The density plots show the frequency of stool consistency across time in days relative to HCT.

Supplementary Figure 4: Timelines of all patients (see Figure 4).

Supplementary Figure 5: Microbiota volatility per day relative to HCT between daily samples (here exclusively $\Delta t = 1$ to avoid any bias that the time scaling may introduce). The line shows a three day rolling average, dashed lines indicate the 95% confidence intervals of the mean.

Supplementary Figure 6: Model predictions. A) Histogram of observed (blue) fold changes in anaerobe counts followed a similar distribution to the posterior predictions (orange). B) Starting from each patient's first observed anaerobe counts, we simulated forwards in time and plot the average predicted anaerobe time course (orange) against the observed (blue).

Supplemental Methods:

Sample preparation and sequencing protocols

DNA extraction: Samples collected for extraction and downstream processing are aliquoted into eppendorf tubes within a 24hour period after the sample was produced. Sample weights are measured and recorded using an electronic balance (Mettler-Toledo). Sample weights are typically 100-500mg depending on how much material was added into the eppendorf tube. Consistency of each sample is recorded as Solid, Semi-Formed, or Liquid. Briefly, a frozen aliquot (≈100 mg) of each sample was suspended, while frozen, in a solution containing 500µl of extraction buffer (200mM Tris, pH 8.0/200mM NaCl/20 mM EDTA), 200µl of 20% SDS, 500µl of phenol:chloroform:isoamyl alcohol (25:24:1), and 500µl of 0.1-mm-diameter zirconia/silica beads (BioSpec Products). Microbial cells were lysed by mechanical disruption with a bead beater (BioSpec Products) for 2 min, after which two rounds of phenol:chloroform:isoamyl alcohol extraction were performed. DNA was precipitated with ethanol at -80 degrees and resuspended in 200µl of TE buffer with 100 mg/ml RNase. The isolated DNA was subjected to additional purification with QIAamp mini spin columns (Qiagen).

16S rDNA amplification and Illumina Sequencing: For each sample, duplicate 50-µl PCR reactions were performed, each containing 50 ng of purified DNA and a master mix of 0.2mM dNTPs, 1.5mM MgCl2, 2.5 U Platinum Taq DNA polymerase, 2.5µl of 10X PCR buffer, and 0.5µM of each primer designed to amplify the V4-V5: 563F (5'-nnnnnnnn-NNNNNNNNNNNN-AYTGGGYDTAAAGNG-3') and 926R (5'- nnnnnnnn-NNNNNNNNNNNN-CCGTCAATTYHTTTRAGT-3'). A unique 12-base Golay barcode (Ns) precede the primers for sample identification [12] and 1-8 additional nucleotides were placed in front of the barcode to offset the sequencing of the primers. Cycling conditions were 94°C for 3 minutes, followed by 27 cycles of 94°C for 50 seconds, 51°C for 30 seconds, and 72°C for 1 minute. 72°C for 5 min is used for the final elongation step. Replicate PCR products were pooled and amplicons were purified using the Qiaquick PCR Purification Kit (Qiagen). PCR products were quantified and pooled at equimolar amounts before proceeding with library preparation following the Illumina TruSeq Sample Preparation protocol. The

completed library was sequenced on an Illumina Miseq platform following the Illumina recommended procedures with a paired end 250 x 250bp kit.

Sequence processing: Paired end reads were assembled, processed, and grouped into operational taxonomic units (OTUs) of 97% similarity using the UPARSE pipeline (13). Sequences were error-filtered, using maximum expected error (Emax=1). Taxonomic assignment to species level was performed for representative sequences form each OUT; this was achieved by using a custom python script incorporating nucleotide BLAST (Basic Local Alignment Search Tool), with the National Center for Biotechnology Information RefSeq as the reference training set (14). We obtained a total of 4,055,808 high-quality 16S rRNA gene-encoding sequences, with a mean of 12,887 sequences per sample. A phylogenetic tree was constructed by aligning representative sequences to SILVA 16S reference.

Sequence designations and identity scores were manually inspected for quality and consistency in terms of taxonomic structure and secondary matches. Based on our testing and comparisons using mock community data, we have found this approach to yield good robust species-level approximations for our candidate sequences. In particular, species-level classification of clostridial species such as Clostridium difficile improved greatly compared with other routine classification methods.

Total 16S quantification: Copy number of 16S rRNA genes for each sample was determined by quantitative PCR (qPCR) on total DNA extracted from fecal samples (s1–s3). Primers specific to the V4 - V5 region of the 16S gene 563F (5′- AYTGGGYDTAAAGNG-3′) and 926Rb (5′- CCGTCAATTYHTTTRAGT-3′) at 0.2 µM concentrations were used with the DyNAmo HS SYBR green qPCR kit (Thermo Fisher Scientific). In order to determine absolute abundances and copy numbers of the 16S gene of unknown samples, a standard was created by taking the V4 and V5 regions from *Escherichia coli* cloned into the *Invitrogen* TOPO pcr2.1 TA vector^{AMP}. The plasmid and insert are 4318bp in length. Copies / µL of our standard is calculated and a total of 7 1:5 serial dilutions starting with 100,000,000 copies create the standard curves which we map our unknown samples against.

The cycling conditions were as follows: 95°C for 10 min, followed by 40 cycles of 95°C for 30 s, 52°C for 30 s, and 72°C for 30 s. 16s qPCR was performed on all stool samples in order to determine bacterial density in feces. We

were unable to amplify bacterial 16S genes from 38 samples, suggesting that bacterial density in these samples was below the level of detection.

Supplementary Bibliography

s1. Tettamanti Boshier F, Srinivasan S, Lopez A, Hoffman NG, Proll S, Fredricks DN, Schiffer JT. 2019. Complementing 16S rRNA gene amplicon sequencing with estimates of total bacterial load to infer absolute species concentrations in the vaginal microbiome. BioRxiv.

s2. Jenq RR, Taur Y, Devlin SM, Ponce DM, Goldberg JD, Ahr KF, Littmann ER, Ling L, Gobourne AC, Miller LC, Docampo MD, Peled JU, Arpaia N, Cross JR, Peets TK, Lumish MA, Shono Y, Dudakov JA, Poeck H, Hanash AM, Barker JN, Perales M-A, Giralt SA, Pamer EG, van den Brink MRM. 2015. Intestinal Blautia Is Associated with Reduced Death from Graft-versus-Host Disease. Biol Blood Marrow Transplant 21:1373–1383.

s3. Liu CM, Prodger JL, Tobian AAR, Abraham AG, Kigozi G, Hungate BA, Aziz M, Nalugoda F, Sariya S, Serwadda D, Kaul R, Gray RH, Price LB. 2017. Penile anaerobic dysbiosis as a risk factor for HIV infection. MBio 8.
